# Supplementary material for: Preventing cardiotoxicity in patients with breast cancer and lymphoma: protocol for a multicentre randomised controlled trial (PROACT)
Source: BMJ Open. 2022 Dec 30;12(12):e066252. doi: 10.1136/bmjopen-2022-066252 (PMC9809245; doi:10.1136/bmjopen-2022-066252)
Supplement: Author's manuscript [file bmjopen-2022-066252.draft_revisions.pdf]

BMJ Open is committed to open peer review. As part of this commitment we make the peer review history of every article we publish publicly available.

When an article is published we post the peer reviewers' comments and the authors' responses online. We also post the versions of the paper that were used during peer review. These are the versions that the peer review comments apply to.

The versions of the paper that follow are the versions that were submitted during the peer review process. They are not the versions of record or the final published versions. They should not be cited or distributed as the published version of this manuscript.

BMJ Open is an open access journal and the full, final, typeset and author-corrected version of record of the manuscript is available on our site with no access controls, subscription charges or pay-per-view fees (http://bmjopen.bmj.com).

If you have any questions on BMJ Open's open peer review process please email info.bmjopen@bmj.com

BMJ Open

Preventing Cardiotoxicity in Patients with Breast Cancer and Lymphoma: protocol for a multi-centre randomised controlled trial (PROACT)

Journal:	*BMJ Open*
Manuscript ID	bmjopen-2022-066252
Article Type:	Protocol
Date Submitted by the Author:	11-Jul-2022

Complete List of Authors:	Maier, Rebecca; Newcastle University, Newcastle Clinical Trials Unit; Academic Cardiovascular Unit, South Tees, South Tees Hospitals NHS Foundation Trust<br>Plummer, Chris; Newcastle Upon Tyne Hospitals NHS Foundation Trust<br>Kasim, Adetayo; Durham University, Durham Research Methods Centre<br>Akhter, Nasima; Durham University, Department of Anthropology<br>Ogundimu, Emmanuel; University of Durham, Mathematical Sciences<br>Maddox, Jamie; Department of Haematology, The James Cook University Hospital, Marton Road, Middlesbrough United Kingdom TS4 3BW, Department of Haematology<br>Graham, Janine; South Tees Hospitals NHS Foundation Trust, Department of Oncology<br>Stewart, Michael; South Tees Hospitals NHS Foundation Trust, Department of Cardiology<br>Wardley, Andrew; Outreach Research & Innovation Group<br>Haney, Sophie; County Durham and Darlington NHS Foundation Trust<br>Vahabi, Sharareh; James Cook University Hospital, Cardiology<br>Oxenham, Helen; North Tees and Hartlepool NHS Foundation Trust<br>Humphreys, Alison; South Tees Hospitals NHS Foundation Trust, Department of Oncology<br>Cresti, Nicola; Newcastle Upon Tyne Hospitals NHS Foundation Trust, Department of Oncology<br>Verrill, Mark; Newcastle Upon Tyne Hospitals NHS Foundation Trust, Department of Oncology<br>Graham, Richard; South Tees Hospitals NHS Foundation Trust<br>Chang, Lisa; South Tees Hospitals NHS Foundation Trust, Academic Cardiovascular Unit, South Tees<br>Hancock, Helen; Newcastle University, Newcastle Clinical Trials Unit<br>Austin, David; James Cook University Hospital

Keywords:	CHEMOTHERAPY, CARDIOLOGY, Breast tumours < ONCOLOGY, Lymphoma < ONCOLOGY, ONCOLOGY

SCHOLARONE™
Manuscripts

For peer review only - http://bmjopen.bmj.com/site/about/guidelines.xhtml

Preventing Cardiotoxicity in Patients with Breast Cancer and Lymphoma: protocol for a multi-centre randomised controlled trial (PROACT)

Chief Investigator: **Dr David Austin**
Consultant Cardiologist
The James Cook University Hospital
South Tees Hospitals NHS Foundation Trust
Marton Road,
Middlesbrough, TS4 3BW
Tel: 01642 850850

Corresponding Author: Rebecca Maier
Affiliations: 1,2.
Email: rebecca.maier@newcastle.ac.uk

Clinical Trials Unit: **Newcastle Clinical Trials Unit**

Funder: National Institute of Health Research – Research for Patient Benefit Programme (project reference PB-PG-0815-20061)

Sponsor: South Tees Hospitals NHS Foundation Trust

Trial Identifiers: ClinicalTrials.gov: NCT03265574
EudraCT: 2017-001094-16
IRAS ID: 213348

Protocol Authors: Maier R 1,2, Plummer C 3, Kasim A 5, Akhter N 5, Ogundimu E 6, J Maddox 7, Graham J 7, Stewart M 8, Wardley A 9, Haney S 10, Vahabi S 8, Oxenham H 11, Humphreys A 7, Cresti N 3, Verrill M 3, Graham R 8, Chang L 2, Hancock H 1 and Austin D 2,8

1. Newcastle Clinical Trials Unit, Newcastle University
2. Academic Cardiovascular Unit, South Tees Hospitals NHS Foundation Trust
3. The Newcastle upon Tyne Hospitals NHS Foundation Trust
4. Centre for Research Methods, Durham University
5. Department of Anthropology, Durham University
6. Department of Mathematical Sciences, Durham University
7. Department of Oncology, South Tees Hospitals NHS Foundation Trust
8. Department of Cardiology, South Tees Hospitals NHS Foundation Trust
9. Outreach Research & Innovation Group
10. County Durham and Darlington NHS Foundation Trust
11. University Hospital of North Tees and Hartlepool

For peer review only - http://bmjopen.bmj.com/site/about/guidelines.xhtml

ABSTRACT – (276/300)

Introduction

Anthracyclines are included in chemotherapy regimens to treat several different types of cancer and are extremely effective. However, it is recognised that a significant side effect is cardiotoxicity; anthracyclines can cause irreversible damage to cardiac cells and ultimately impaired cardiac function and heart failure which may only be evident years after exposure. The PROACT trial will establish the effectiveness of the angiotensin-converting enzyme inhibitor (ACEI) enalapril maleate (enalapril) in preventing cardiotoxicity in patients with breast cancer and Non-Hodgkin Lymphoma (NHL) receiving anthracycline-based chemotherapy.

Methods and Analysis

PROACT is a prospective, randomised, open-label, blinded end-point, superiority trial which will recruit adult patients being treated for breast cancer and NHL at NHS hospitals throughout England. The trial aims to recruit 106 participants, who will be randomised to standard care (high dose anthracycline-based chemotherapy) plus enalapril (intervention), or standard care alone (control). Patients randomised to the intervention arm will receive enalapril (starting at 2.5 mg bd and titrating up to a maximum dose of 10mg bd), commencing treatment at least two days prior to starting chemotherapy and finishing three weeks after their last anthracycline dose. The primary outcome is the presence or absence of cardiac troponin T release at any time during anthracycline treatment, and one month after the last dose of anthracycline. Secondary outcomes will focus on cardiac function measured using echocardiogram assessment, adherence to enalapril, and side effects.

Ethics and Dissemination

A favourable opinion was given following Research Ethics Committee review by West Midlands - Edgbaston REC. Trial findings will be disseminated through engagement with patients, the oncology and cardiology communities, NHS management and commissioning groups, and through peer reviewed publication.

Trial Registration Number

ClinicalTrials.gov: NCT03265574

Strengths and Limitations of this Trial

1. Strong and ongoing patient and public involvement (PPI) and clinical consensus informed the trial design.
2. Robust randomised trial design, with 80% power.
3. Multi-centre UK trial with blinding of the primary outcome and the echo core laboratory reduces bias and will enable robust findings.

For peer review only

For peer review only - http://bmjopen.bmj.com/site/about/guidelines.xhtml

4. The intervention is low cost, and with decades of evidence indicating its safety; if findings from PROACT demonstrate effectiveness, its translation into patient benefit can be realised through rapid adoption into usual care.
5. Findings will have implications for multiple cancers across adult and paediatric settings where patients receive high dose anthracycline chemotherapy.

Introduction

Breast cancer is the most common malignancy among women worldwide, with over 55,000 new UK cases every year[1,2]. Lymphoma is the most common haematological malignancy and the sixth most common cancer in the UK, with Non-Hodgkin Lymphoma (NHL) affecting 14,000 patients per year[3]. Treatment for both conditions includes anthracycline chemotherapy (epirubicin or doxorubicin); however, anthracyclines can cause immediate, irreversible damage to cardiac cells and ultimately impaired cardiac function and heart failure which may only be evident years after exposure.[4] The long-term incidence is approximately 5% and may be higher in older patients.[5,6,7] Asymptomatic left ventricular systolic dysfunction (LVSD) is more common, evident in 9.7% of patients with breast cancer within 12 months.[8.] LVSD affects cardiac prognosis and limits subsequent cancer treatment options, particularly those that target the HER2 pathway such as trastuzumab (Herceptin).[8,9] In lymphoma, due to significant improvements in survival rates, there is also greater prevalence of anthracycline-induced cardiotoxicity in survivors.[10] Waiting until anthracycline-induced heart failure becomes clinically evident is ill-advised, with response to treatment poor and survival at 2 years of 40%; considerably worse than other causes of heart failure.[11]

The burden of cardiotoxicity is particularly important in both patient groups where 78% (breast) and 63% (NHL) will be alive 10 years following their cancer diagnosis.[1,12] Preventing cardiac damage would offer clear benefits to patients and substantial costs savings to the NHS.

Current Evidence Supporting the Rationale for the Trial

Anthracycline toxicity is thought to be due to the generation of reactive oxygen species causing cell death.[13,14] ACEI promote nitric oxide and inhibit the production of angiotensin II, which in turn reduces NAD(P)H oxidase responsible for superoxide formation. ACEI and angiotensin inhibition are protective of apoptosis in vascular endothelium and cardiac cells, prevent anthracycline cardiotoxicity in animal models and reduce oxidative stress.[15-19]

ACEI are of proven benefit in cardiovascular medicine, and are widely used, well tolerated and inexpensive (cost £2 per patient per month). Enalapril is the reference standard ACEI and has been extensively studied.[20, 21]

Treatment to prevent cardiotoxicity is attractive and feasible but is unproven. A Cochrane systematic review was unable to make definitive conclusions regarding the effectiveness of previously studied

For peer review only - http://bmjopen.bmj.com/site/about/guidelines.xhtml

cardioprotective agents; no studies of ACEI were included.[22] A meta-review and further systematic review also found a lack of evidence to guide decision-making.[23,24] A European Society of Cardiology position paper also came to the same conclusion on preventative therapy.[25]

An Italian study assessed enalapril treatment in patients with a positive troponin during chemotherapy (not all were anthracycline-based regimens). By the second month, 41% of patients in the control group (n=58) had persistent troponin elevation, compared to 4% in the enalapril group (n=56). Based on echocardiography, no cardiotoxicity in the enalapril group was observed; cardiotoxicity was identified in 43% of controls ($p<0.05$).[26]

Small European studies assessed preventative strategies in lower doses of anthracyclines (average 240 mg/m^2), but demonstrated that LVEF measured by cardiac MRI declined less in patients treated with candestartan[27]; similar results are seen in those treated with enalapril and carvedilol.[28] Subsequent 2 year follow up of the PRADA study suggested only a small decline in LVEF in patients receiving lower dose anthracyclines, and no significant between group differences.[29]

ICOS ONE randomised patients to enalapril or “troponin-triggered” enalapril and found no difference between the two strategies. Median dose of anthracyclines was low (180mg/m^2) and the most enalapril was typically prescribed at 2.5 mg bd. The rate of cardiotoxicity at 3 years was low.[30,31]

A UK based clinical trial, CARDIAC CARE (ISRCTN24439460), is testing a different hypothesis to PROACT; patients with breast cancer or NHL will have troponin-guided randomisation to treatment with beta-blockers and ACEI or usual care. CARDIAC CARE is currently recruiting and will provide complimentary information to PROACT on its completion.[32]

Troponin T is a sensitive marker of early cardiac cell death and as such there is the potential for troponin release to be “turned off” if enalapril is effective. Audit data from 36 patients and 143 samples, showed a positive troponin T in 47% of patients who received >300 mg/m^2 of anthracycline in a six-cycle regimen. Troponin T was chosen as the primary outcome measure on this basis, and was endorsed by our patient groups. It is known from previous research that troponin correlates with subsequent changes in LV function, and importantly that a negative troponin during and at one month post chemotherapy essentially excludes significant cardiotoxicity.[33,34]

There are currently no definitive trials of ACEI in the prevention of anthracycline cardiotoxicity in patients receiving the highest contemporary doses of chemotherapy. PROACT will determine the effectiveness of enalapril in preventing cardiotoxicity in patients receiving high-dose anthracycline-based chemotherapy for breast cancer and NHL. Results will also inform practice for other cancer types. Findings will directly inform clinical practice in confirming whether enalapril should be given routinely to patients receiving anthracycline-based chemotherapy for breast cancer and NHL.

Increasing breast cancer and NHL survival, the frequency and impact of cardiotoxicity and the potential

For peer review only - http://bmjopen.bmj.com/site/about/guidelines.xhtml

for a simple, safe and cheap preventative treatment makes PROACT highly important for patients and the NHS.

METHODS AND ANALYSIS

Study Design

PROACT is a prospective, randomised, open-label, blinded end-point, superiority trial in patients undergoing anthracycline-based chemotherapy for breast cancer or NHL. Patients will be randomised to receive enalapril (intervention) or control (standard care).

The trial will answer the question 'Can troponin release be prevented by enalapril in patients undergoing high dose anthracycline chemotherapy treatment for cancer?

Setting

Patients due to receive high dose anthracycline-based chemotherapy for their breast cancer or NHL at participating NHS Trusts will be offered recruitment to the trial. All sites can accommodate the needs of this trial including research nurse support, facilities for trial interventions and assessments, and British Society of Echocardiography (BSE) accredited echocardiographers, advanced trainee, or consultant cardiologists, to carry out echocardiograms in accordance with the trial protocol.

Eligibility Criteria

Inclusion criteria:

- Adult patients with histopathologically* confirmed breast carcinoma who have received surgery for their breast cancer;
- Planned to receive 6 cycles of **EC 90** (total planned dose 540 mg/m^2 epirubicin) or **FEC 75** (total planned dose 450 mg/m^2 epirubicin) adjuvant chemotherapy regimen;

OR

- Adult patients with histopathologically confirmed non-Hodgkin lymphoma planned to receive 6 cycles of **R-CHOP or CHOP** (total planned dose 300mg/m^2 doxorubicin) chemotherapy**

AND

- Written informed consent.

**Patients with HER2+ breast cancer are eligible for inclusion.*

***Patients who will receive an alternative anti-CD20 monoclonal antibody are eligible (for example O-CHOP), as long as the total planned doxorubicin dose is ≥300mg/m^2 over 6 cycles*

Exclusion criteria:

- positive baseline cardiac troponin T (≥14 ng/L);
- known contraindication to ACE inhibitor e.g. renal artery stenosis, severe aortic stenosis;
- are taking, or have a previous intolerance to ACEI (e.g. angioedema);

For peer review only - http://bmjopen.bmj.com/site/about/guidelines.xhtml

- patient already taking other agents acting on the renin-angiotensin-aldosterone system e.g., Aliskiren, angiotensin receptor blockers (ARBs), Entresto (sacubitril/valsartan), spironolactone, eplerenone;
- LVEF <50%;
- estimated GFR < 30 mL/min/1.73m^2 at baseline;
- hyperkalaemia defined as serum potassium ≥5.5mmol/L;
- symptomatic hypotension, or Systolic Blood Pressure <100mmHg;
- poorly-controlled hypertension (Blood Pressure >160/100mmHg, or ambulatory BP of 150/95mmHg);
- previous myocardial infarction;
- known metastatic breast cancer;
- previous exposure to anthracycline chemotherapy;
- are pregnant or breastfeeding;
- previous trastuzumab treatment or planned trastuzumab treatment within four weeks following anthracycline chemotherapy;
- for patients of childbearing potential: refusal to use adequate contraception throughout trial;***
- any other invasive cancer diagnosed and treated in the past 5 years;
- symptomatic or severe asymptomatic radiation-induced cardiac disease;
- participation in other interventional medicinal trials in the past 6 months;
- judgement by the investigator that the patient has a prognosis of < 1 year or are unlikely to complete 6 cycles of chemotherapy.
- judgement by the investigator that the patient is high risk for tumour lysis syndrome (applicable only to NHL patients).
- judgement by the Investigator that the patient should not participate in the study, (e.g., if patient is unlikely to comply with study procedures, restrictions, and requirements.

***Female patients between the ages of 18 and 50 will receive a pregnancy test at baseline.

Randomisation

Randomisation will use a minimisation scheme which adjusts for baseline factors; the minimisation scheme will account for the planned 6 cycle chemotherapy regimen (EC 90 or FEC 75, or R-CHOP), and, in breast cancer HER2 status (positive or negative). Patients will be randomised 1:1 to either standard care plus enalapril, or standard care only by members of the research team at each centre using a 24-hour, central, secure, web-based randomisation system with concealed allocation (procured from Sealed Envelope Ltd).

Trial Intervention

Standard Care

For peer review only - http://bmjopen.bmj.com/site/about/guidelines.xhtml

All patients in both arms will be planned to receive 6 cycles of chemotherapy as part of standard care. There is no placebo in the standard care alone arm.

The following regimens are permitted within the trial:

Breast Cancer regimens:

- EC 90; Epirubicin 90mg/m^2, Cyclophosphamide 600mg/m^2
- FEC 75; Fluorouracil 600mg/m^2, Epirubicin 75mg/m^2, Cyclophosphamide 600mg/m^2

Non Hodgkin Lymphoma regimens:

- R-CHOP* or CHOP; (Rituximab 375mg/m^2), Cyclophosphamide 750mg/m^2, Doxorubicin 50mg/m^2, Vincristine 1.4mg/m^2 (max 2mg), Prednisolone 40mg/m^2 (for 5 days).

**Patients who receive an alternative anti-CD20 monoclonal antibody are eligible (for example O-CHOP), as long as the total planned doxorubicin dose is ≥300mg/m^2 over 6 cycles*

Intervention arm

Patients randomised to the intervention arm will receive enalapril in addition to standard care. Patients will commence on a 2.5mg bd dose at least two days prior to their first dose of anthracycline chemotherapy, with the aim to up-titrate the dose to 5mg bd, then 10mg bd over a maximum of three dose evaluation assessments prior to cycle 2. Chemotherapy will not be delayed by taking part in the trial. Some clinicians/centres routinely withhold ACEI on the morning of rituximab therapy; this is allowed per protocol.

Dose evaluation assessment visits will take place between two and seven days after the start of each dose level. If the patient has systolic BP ≥100 mmHg, normal serum potassium (potassium <5.5 mmol/L), and stable renal function (serum creatinine <30 μmol/L) the dose will be increased to the next dose level. If clinical opinion is that the patient is unlikely to tolerate the higher dose, the dose will remain the same. If the patient has a ≥30 μmol/L increase in serum creatinine levels since the last assessment compared to baseline, new hyperkalaemia (potassium ≥5.5mmol/L), or symptomatic hypotension (SBP <100mmHg) then enalapril will be permanently discontinued. Patients will remain on the maximum enalapril dose reached for the duration of their chemotherapy, and until three weeks following their last dose of anthracycline. Temporary halts of up to 14 days will be allowed, with re-introduction and dose reductions at the discretion of the Investigator. Extensive PPI work at the point of trial design, and financial cost, led to a decision not to include a placebo in the control arm.

Blinding

PROACT is an open label trial. The trial employs a prospective randomised blinded endpoint design; analysis of troponin T and troponin I will be completed by laboratory staff who are blinded to the patients' trial allocation, as detailed in the trial PROACT laboratory manual. The trial management team, statistics, and clinical teams will remain blinded to the troponin results until the end of the trial. The Data Manager, and an un-blind monitor separate to the trial team, will have access to the troponin results for the purposes of monitoring and data cleaning.

For peer review only - http://bmjopen.bmj.com/site/about/guidelines.xhtml

All echocardiograms will be sent to an independent Core Laboratory for assessment by a BSE accredited echocardiographer/or an advanced trainee or consultant cardiologist blind to the intervention.

Outcomes

Primary outcome:

The primary outcome is cardiotoxicity measured as presence (≥14ng/L) of cardiac troponin T release at any time during anthracycline treatment, and one month after the last dose of anthracycline.

Secondary outcomes:

- Cardiac function will be assessed by echocardiogram, including global longitudinal strain (GLS), and measurements of left ventricular ejection fraction (LVEF), at baseline and following completion of chemotherapy;
- Cardiotoxicity will be measured as cardiac troponin I release during chemotherapy and at one month after the last dose of anthracycline;
- Adherence to enalapril will be measured according to patient diaries throughout the trial;
- All adverse events, and adverse reactions including those that are serious and unexpected will be recorded from the day of randomisation until the last visit or until withdrawal; adverse events considered related to enalapril will be followed until resolution, a stable outcome or death.
- Anxiety or distress related to trial participation will be measured at the last study visit;
- Cancer and chemotherapy outcomes will be characterised in the population.

Protocol Changes

Breast cancer management

Shortly after the trial opened to recruitment, changes in the clinical management of patients with breast cancer reduced the number of patients potentially eligible patients. The first was the widespread adoption of genomic testing to calculate the Oncotype Dx recurrence score, which reduced the number of patients recommended for adjuvant chemotherapy by approximately 30% in our recruiting hospitals.

In addition, in July 2018 NICE published a clinical guideline on the management of breast cancer which included recommending a taxane to be routinely offered alongside an anthracycline in adjuvant chemotherapy (FEC-T chemotherapy); in the development of PROACT, we identified patients receiving FEC-T as being at low risk of cardiotoxicity. It was decided to include NHL patients in the trial to increase the number of potentially eligible patients.

COVID 19 impact

PROACT clinical trial delivery has been significantly affected by the COVID 19 pandemic. The trial was paused to recruitment in March 2020, and whilst it has re-opened to recruitment, ongoing impact on non-

For peer review only - http://bmjopen.bmj.com/site/about/guidelines.xhtml

COVID research in the NHS, and in particular recruitment to Oncology clinical trials, is well documented. NIHR have agreed two funded extensions to enable the study to complete.

Sample Size

In light of the unprecedented challenges, the trial team, in agreement with funder, Trial Steering Committee (TSC) and Independent Data Monitoring and Ethics Committee (IDMEC) agreed to recalculate the sample size by changing from 90% to 80% power, using the same assumptions as in the original sample size calculation.

Assuming alpha of 5%, and 80% power, 106 patients are needed to detect a reduction in the proportion of patients with cardiac troponin T present from 47% to 20% using a two-sided Fisher's exact test; additional recruitment to account for attrition is planned. Observational data was used to provide us with the estimate of troponin T elevation in the standard care arm; there are other potential causes of an elevated troponin, such as infection, during chemotherapy and clinical consensus indicated that a rate of 20% in elevated troponin would fully account these. There is also a consensus within the clinical community that, a large effect size will be necessary to convince the clinical community to change the pathway of care for these patients. The original sample size at 90% power was 140 patients, inflated to 170 patients to account for attrition.

Trial Procedures

Patients due to undergo 6 cycles of anthracycline based chemotherapy for breast cancer or NHL at participating centres are identified by the clinical research team and approached about the trial, including given an information sheet and consent form. After discussion, consent is sought, baseline assessments performed and eligibility checked and confirmed. Eligible patients are randomised, and their General Practitioners informed. A full schedule of events is detailed in Table 1, and a participant flow chart provided in Figure 1. Patients have assessments at baseline, dose evaluation visits (up to three for those receiving the intervention) within 72 hours prior to day 1 of each chemotherapy cycle, and at trial completion (4 weeks following the last dose of anthracycline). A separately funded study is allowing patients to be followed for one year, and all patients are asked to consent to longer term follow up.

Baseline activities include consent, medical history, height/weight, NYHA class, BP check, bloods, eGFR and eligibility check.

For peer review only - http://bmjopen.bmj.com/site/about/guidelines.xhtml

For peer review only

Table 1: PROACT Schedule of Events

	Baseline (up to 6 weeks prior to randomisation)	Day 1 (at least 2 days prior to Cycle 1 Day 1)	Dose evaluation visit 1	Dose evaluation visit 2	Dose evaluation visit 3	Cycle 1, Day 1	Cycle 2, Day 1	Cycle 3, Day 1	Cycle 4, Day 1	Cycle 5, Day 1	Cycle 6, Day 1	Trial Completion Visit	1-year Follow-up Visit
Consent	X												
Demographics	X												
Medical History	X												X
Cancer history	X												X
Performance Status	X												X
NYHA Class	X												X
Concomitant medications	X	X	X	X	X	X	X	X	X	X	X	X	X
Physical Assessment (height and weight)	X												X
Blood Pressure	X		X	X	X	X	X	X	X	X	X	X	X
Troponin T (baseline sample)	X												
Troponin T (post-baseline)							X	X	X	X	X	X	
Troponin I	X						X	X	X	X	X	X	
U+Es	X		X	X	X		X	X	X	X	X	X	
eGFR	X												
Pregnancy test	X												
Echocardiogram	X											X	X
Eligibility check	X												
Randomisation	X												
Enalapril (intervention group only)		X	X	X	X	X	X	X	X	X	X		
Adherence to enalapril (intervention group only)		X	X	X	X	X	X	X	X	X	X	X	
Standard care chemotherapy (all patients)						X	X	X	X	X	X		
Adverse Events		X	X	X	X	X	X	X	X	X	X	X	
Buccal swab for future research	X												
Blood sample for future research	X							X		X		X	
Acceptability of trial interventions assessment									X			X	

For peer review only - http://bmjopen.bmj.com/site/about/guidelines.xhtml

Troponin T and Troponin I

Blood sampling for troponin T and I will be performed at baseline, within 72 hours prior to chemotherapy (Cycle 2 onwards), and trial completion (30 days after the final anthracycline dose). Troponin T samples will provide the primary outcome data. Samples will be processed and sent to a core laboratory for central analysis; the central laboratory will be blind to treatment allocation.

Echocardiogram

Cardiac function is assessed via transthoracic echocardiography (TTE) at baseline, four weeks after the last anthracycline dose and one year after the date of final chemotherapy. All echocardiograms will be assessed by a Core Laboratory who will be blind to participant treatment. Local reporting of TTEs will be as per local hospital practice; the data reported by the Core Laboratory (including GLS and LVEF) will be analysed as part of the secondary outcomes for the trial.

Trial Anxiety or Distress Participation Assessment

At the start of cycle 4 and at the end of study visit (1 month after the last chemotherapy treatment), patients will be asked to complete a short questionnaire to understand if taking part in the trial has resulted in any anxiety or distress. This outcome was requested by the funding panel and will help to understand the impact on patients of a change to standard care in the event that PROACT demonstrates the effectiveness of enalapril in this setting.

Statistical Analysis

Data cleaning and analysis will be provided by staff within NCTU and Durham University. Primary analysis will follow intention to treat principles with patient data analysed according to randomisation and irrespective of intervention received; other analysis groups such as per-protocol may be considered subsequently. Every effort will be made to retain and include all patients who are part of the trial.

A full statistical analysis plan (SAP) will be developed for the outcome measures and agreed with the Independent Data Monitoring and Ethics Committee (IDMEC) and Chief Investigator prior to any analysis being undertaken.

Outcome data will be analysed at the end of the main study, no interim analysis is planned. Follow-up data will be analysed and reported separately.

The primary analysis of presence or absence of cardiac troponin T will be assessed using logistic regression and accounting for minimisation factors. Analysis of the secondary endpoints will be dependent upon the nature of the specific endpoint and data structure; global longitudinal strain (GLS) and left ventricular ejection fraction (LVEF) will be analysed as a change from baseline using regression model to compare intervention and control groups.

Cardiac troponin I data will also be analysed using logistic regression and account for minimisation factors. Changes in cardiac troponin T and I will also be analysed as a continuous variable. Additional analysis of the primary endpoint will be performed using logistic regression to account for baseline

For peer review only - http://bmjopen.bmj.com/site/about/guidelines.xhtml

factors (regimen and HER2 status). Adverse events data will be analysed using cross-tabulation. Sensitivity analysis will also be performed for adherence to the protocol and compare breast cancer and non-breast cancer patients.

Trial Conduct and governance

The Trial Management Group is responsible for the day to day management of the trial, overseeing all aspects to ensure that the protocol is adhered to and taking appropriate actions to ensure patient safety and data integrity. The IDMEC review trial outcomes (including adverse events and serious adverse events), provide advice on the ongoing conduct and safety of the trial and report recommendations to the TSC. The TSC, where independent members are the majority (including patient representatives), provides overall supervision, monitors progress and conduct and advises on the trial. The IDMEC and TSC will meet every six months.

Patient and Public Involvement

Initial trial ideas were discussed with patients who had recently finished chemotherapy treatment for breast cancer the Maggie's Centre in Newcastle in 2015 and at a breast cancer support group in North Yorkshire and again with patients from the Maggie's Centre; trial ideas were developed between each discussion based on their feedback prior to submission of the application.

The trial also has two patient representatives who sit on the Trial Steering Committee; one patient previously treated for breast cancer, the other previously treated for NHL. Neither of these patients are trial participants, nor are they employed by any organisation directly involved in the trial conduct.

Ethics and dissemination

All clinical research raises ethical issues, and the trial team carefully thought through potential issues and aimed to address these in the trial design. This study does not include patients who will lack capacity, nor minors. This trial is not based in an emergency setting. The trial is conducted in accordance with the protocol, Good Clinical Practice, the favourable ethical opinion and the MHRA notice of no-objection. All patients provide written informed consent prior to participation. Enalapril has been widely used for over thirty years, is well tolerated without significant side effects.

If this research demonstrates that enalapril is effective in preventing heart damage in patients receiving anthracycline chemotherapy this will have a significant impact not just for patients with breast cancer and NHL, but for both adult and paediatric patients with other types of cancer treated with chemotherapy regimens containing anthracyclines. In addition to reduced morbidity and mortality for patients, enalapril will provide significant savings for the NHS.

We will publish the findings in peer-reviewed journals, and disseminate results to patients, and the international clinical community.

For peer review only - http://bmjopen.bmj.com/site/about/guidelines.xhtml

Acknowledgements

We are grateful to members of the IDMEC, and TSC for their support of PROACT. We would like to acknowledge the considerable work being undertaken by Principal Investigators and members of the site teams in support of this trial, and the PROACT trial team at Newcastle Clinical Trials Unit.

Authors Contributions

DA and CJP conceived the idea for the trial. DA, HCH, RHM, ASK, CJP, MV, NC, JG, JM, MS, AW, SH, HO, AH, RG co-designed the trial, secured funding from the NIHR RfPB and wrote the full trial protocol with substantial input from LC. DA is the Chief Investigator; RHM and HCH provide methodological input and oversee NCTU activity. EO leads the statistical aspects and analysis, overseeing NA. SV undertakes echocardiogram review and reporting at the core echocardiogram laboratory. This paper was drafted from the approved version of the protocol; all authors commented and amended drafts of the paper and approved the final manuscript.

Funding Statement

This work is supported by the National Institute for Health Research (NIHR) Research for Patient Benefit (RfPB) Programme (Ref: PB-PG-0815-20061).

The PROACT Trial is sponsored by South Tees Hospitals NHS Foundation Trust.

Conflicts of Interest

Professor Adetayo Kasim's contribution was during his employment by Durham University. He currently works for UCB Biopharma, UK.

Professor Andrew Wardley currently works for Outreach Research & Innovation Group, and was employed by The Christie NHS Foundation Trust, Manchester when the grant was awarded.

Dr David Austin has previously received speaker fees from Astra Zeneca, Pfizer and Philips/Volcano. None were directly relevant to PROACT

David Austin https://orcid.org/0000-0003-4606-1055

Rebecca Maier: https://orcid.org/0000-0002-7350-3288

Helen Hancock: https://orcid.org/0000-0002-1494-8551

Adetayo Kasim: https://orcid.org/0000-0002-0411-3059

Nicola Cresti: https://orcid.org/0000-0002-0495-4472

Jamie Maddox: https://orcid.org/0000-0002-7995-7934

Nasima Akhter: https://orcid.org/0000-0002-5424-1593

Emmanuel Ogundimu: https://orcid.org/0000-0001-9252-9275

Andrew Wardley: https://orcid.org/0000-0002-9639-0888

Licence Statement

I, the Submitting Author has the right to grant and does grant on behalf of all authors of the Work (as defined in the below author licence), an exclusive licence and/or a non-exclusive licence for contributions from authors who are: i) UK Crown employees; ii) where BMJ has agreed a CC-BY licence shall apply,

For peer review only - http://bmjopen.bmj.com/site/about/guidelines.xhtml

and/or iii) in accordance with the terms applicable for US Federal Government officers or employees acting as part of their official duties; on a worldwide, perpetual, irrevocable, royalty-free basis to BMJ Publishing Group Ltd ("BMJ") its licensees and where the relevant Journal is co-owned by BMJ to the co-owners of the Journal, to publish the Work in BMJ Open and any other BMJ products and to exploit all rights, as set out in our licence.

The Submitting Author accepts and understands that any supply made under these terms is made by BMJ to the Submitting Author unless you are acting as an employee on behalf of your employer or a postgraduate student of an affiliated institution which is paying any applicable article publishing charge ("APC") for Open Access articles. Where the Submitting Author wishes to make the Work available on an Open Access basis (and intends to pay the relevant APC), the terms of reuse of such Open Access shall be governed by a Creative Commons licence – details of these licences and which Creative Commons licence will apply to this Work are set out in our licence referred to above.

For peer review only

REFERENCES

1. Cancer Research UK. Breast cancer statistics.http://www.cancerresearchuk.org/health-professional/cancer-statistics/statistics-by-cancer-type/breast-cancer. Accessed 26th April 2022.
2. World Cancer Research Fund International. Breast cancer statistics. https://www.wcrf.org/cancer-trends/breast-cancer-statistics/ Accessed 26th April 2022.
3. Cancer Research UK Non-Hodgkin Lymphoma Statistics https://www.cancerresearchuk.org/health-professional/cancer-statistics/statistics-by-cancer-type/non-hodgkin-lymphoma Accessed 26th April 2022.
4. Groarke JD, Nohria A. Anthracycline Cardiotoxicity: A New Paradigm for an Old Classic. Circulation 2015;**131**(22):1946-9.
5. Swain SM, Whaley FS, Ewer MS. Congestive heart failure in patients treated with doxorubicin: a retrospective analysis of three trials. Cancer 2003;**97**(11):2869-79.
6. Pinder MC, Duan Z, Goodwin JS*, et al.* Congestive heart failure in older women treated with adjuvant anthracycline chemotherapy for breast cancer. J Clin Oncol 2007;**25**(25):3808-15.
7. Steinherz LJ, Steinherz PG, Tan CC*, et al.* Cardiac toxicity 4 to 20 years after completing anthracycline therapy. JAMA 1991;**266**(12):1672-77.
8. Cardinale D, Colombo A, Bacchiani G*, et al.* Early Detection of Anthracycline Cardiotoxicity and Improvement With Heart Failure Therapy. Circulation 2015;**131**(22):1981-8.
9. Piccart-Gebhart MJ, Procter M, Leyland-Jones B*, et al.* Trastuzumab after Adjuvant Chemotherapy in HER2-Positive Breast Cancer. N Engl J Med 2005;**353**(16):1659-72.
10. Ewer M, Ewer S. Cardiotoxicity of anticancer treatments. Nature Reviews Cardiology. 2015;**12**(9):547-558.
11. Felker GM, Thompson RE, Hare JM*, et al.* Underlying Causes and Long-Term Survival in Patients with Initially Unexplained Cardiomyopathy. N Engl J Med 2000;**342**(15):1077-84.
12. Quaresma M, Coleman MP, Rachet B. 40-year trends in an index of survival for all cancers

For peer review only - http://bmjopen.bmj.com/site/about/guidelines.xhtml

combined and survival adjusted for age and sex for each cancer in England and Wales, 1971-2011: a population-based study. Lancet 2015;**385**(9974):1206-18.

13. Münzel T, Keaney JF. Are ACE Inhibitors a “Magic Bullet” Against Oxidative Stress? Circulation 2001;**104**(13):1571-74.
14. Rajagopalan S, Politi PM, Sinha BK*, et al.* Adriamycin-induced free radical formation in the perfused rat heart: implications for cardiotoxicity. Cancer Res 1988;**48**(17):4766-9.
15. Vaynblat M, Shah HR, Bhaskaran D*, et al.* Simultaneous angiotensin converting enzyme inhibition moderates ventricular dysfunction caused by doxorubicin. Eur J Heart Fail 2002;**4**(5):583-6.
16. Sacco G, Mario B, Lopez G*, et al.* ACE inhibition and protection from doxorubicin-induced cardiotoxicity in the rat. Vascul Pharmacol 2009;**50**(5-6):166-70.
17. Noda K, Kobara M, Hamada J*, et al.* Additive amelioration of oxidative stress and cardiac function by combined mineralocorticoid and angiotensin receptor blockers in postinfarct failing hearts. J Cardiovasc Pharmacol 2012;**60**(2):140-9.
18. Ferrari R, Fox K. Insight into the Mode of Action of ACE Inhibition in Coronary Artery Disease. Drugs 2009;**69**(3):265-77.
19. Galvez AS, Fiedler JL, Ocaranza MP*, et al.* Perindopril regulates beta-agonist-induced cardiac apoptosis. J Cardiovasc Pharmacol 2005;**46**(3):255-61.
20. McMurray JJV, Packer M, Desai AS*, et al.* Angiotensin–Neprilysin Inhibition versus Enalapril in Heart Failure. N Engl J Med 2014;**371**(11):993-1004.
21. British National Formulary. https://www.evidence.nhs.uk/formulary/bnf/current Accessed 28th November 2015.
22. van Dalen EC, Caron HN, Dickinson HO*, et al.* Cardioprotective interventions for cancer patients receiving anthracyclines. Cochrane Database Syst Rev 2011(6):CD003917.
23. Kalam K, Marwick TH. Role of cardioprotective therapy for prevention of cardiotoxicity with chemotherapy: a systematic review and meta-analysis. Eur J Cancer 2013;**49**(13):2900-9.
24. Conway A, McCarthy AL, Lawrence P*, et al.* The prevention, detection and management of cancer treatment-induced cardiotoxicity: a meta-review. BMC Cancer 2015;**15**(1):366.
25. Zamorano JL, Lancellotti P, Rodriguez Muñoz D*, et al.* 2016 ESC Position Paper on cancer treatments and cardiovascular toxicity developed under the auspices of the ESC Committee for Practice Guidelines. The Task Force for cancer treatments and cardiovascular toxicity of the European Society of Cardiology (ESC) 2016;**37**(36):2768-2801
26. Cardinale D, Sandri MT, Colombo A*, et al.* Prognostic value of troponin I in cardiac risk stratification of cancer patients undergoing high-dose chemotherapy. Circulation 2004;**109**(22):2749-54
27. Gulati G, Heck SL, Ree AH*, et al.* Prevention of cardiac dysfunction during adjuvant breast cancer therapy (PRADA): a 2 × 2 factorial, randomized, placebo-controlled, double-blind clinical trial of candesartan and metoprolol. European Heart Journal 2016;**37**(21):1671-80
28. Bosch X, Rovira M, Sitges M*, et al.* Enalapril and carvedilol for preventing chemotherapy-induced left ventricular systolic dysfunction in patients with malignant hemopathies: the OVERCOME trial (preventiOn of left Ventricular dysfunction with Enalapril and caRvedilol in

For peer review only - http://bmjopen.bmj.com/site/about/guidelines.xhtml

patients submitted to intensive ChemOtherapy for the treatment of Malignant hEmopathies). J Am Coll Cardiol 2013;**61**(23):2355-62.

29. Lagethon Heck S, Hansen Rec A, Mecinaj A, *et al.* Prevention of cardiac dysfunction during adjuvant breast cancer therapy (PRADA): Extended follow up of a 2x2 factorial, randomised, placebo-controlled, double blind trial of candesartan and metoprolol. Circulation 2021; **143** (25): 2431-2440
30. Cardinale D, Ciceri F, Latini R, *et al*. Anthracycline-induced cardiotoxicity: A multicenter randomised trial comparing two strategies for guiding prevention with enalapril: The International CardioOncology Society-one trial. Eur J Cancer 2018 **94**:126-137.
31. Meessen JMTA, Cardinale D, Ciceri F, *et al*. Circulating biomarkers and cardiac function over 3 years after chemotherapy with anthracyclines: the ICOS-ONE trial. ESC Heart Fail. 2020; **7**(4):1452-1466.
32. CARDIAC CARE The Cardiac Care Trial. http://www.isrctn.com/ISRCTN24439460 accessed 26th April 2022.
33. Cardinale D, Sandri MT, Martinoni A*, et al.* Left ventricular dysfunction predicted by early troponin I release after high-dose chemotherapy. J Am Coll Cardiol 2000;**36**(2):517
34. Cardinale D, Sandri MT, Colombo A*, et al.* Prognostic value of troponin I in cardiac risk stratification of cancer patients undergoing high-dose chemotherapy. Circulation 2004;**109**(22):2749-54.

Figure 1: PROACT Trial Flow Diagram

For peer review only - http://bmjopen.bmj.com/site/about/guidelines.xhtml

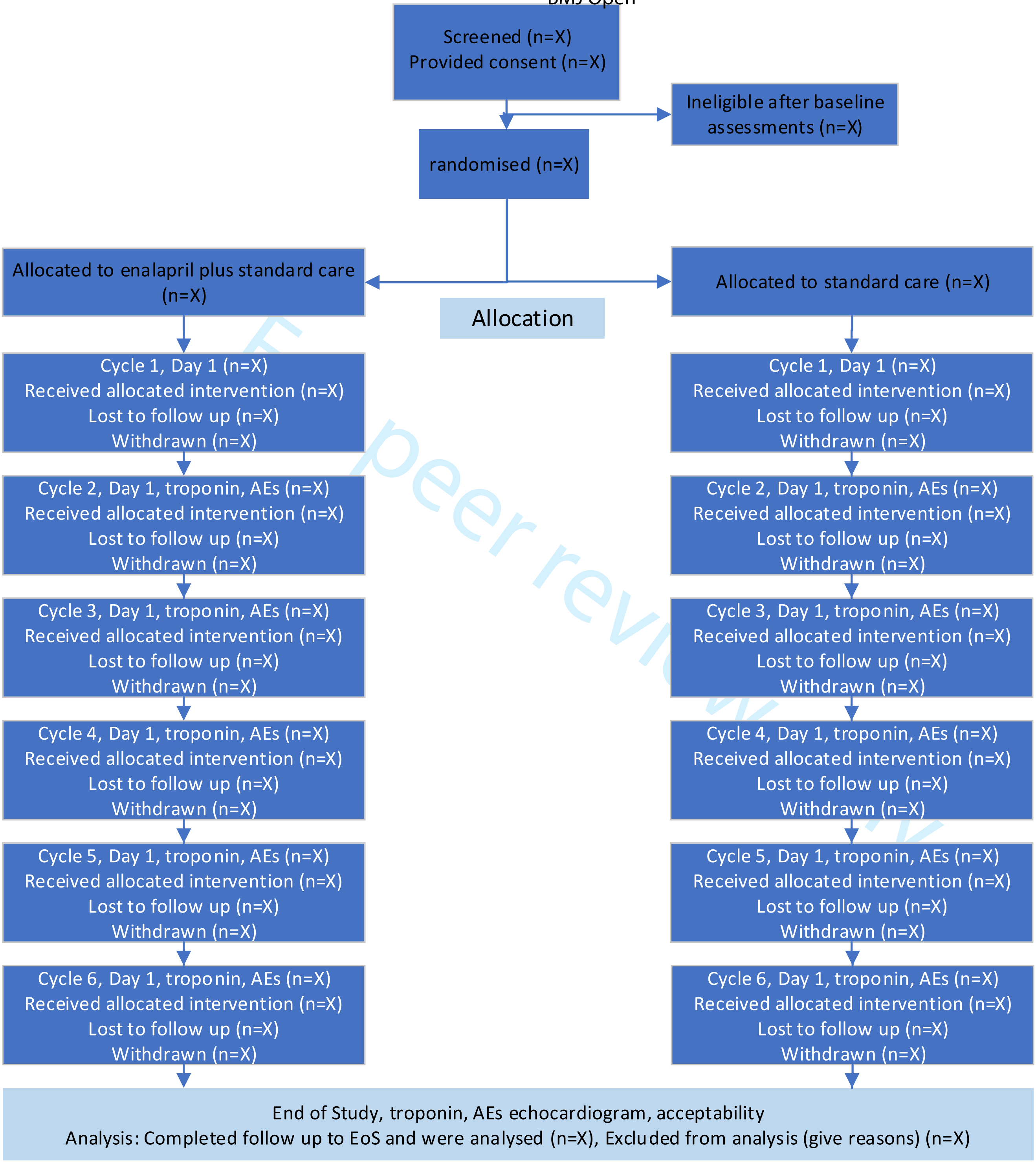
Screened (n=X)
Provided consent (n=X)
Ineligible after baseline assessments (n=X)
randomised (n=X)
Allocated to enalapril plus standard care (n=X)
Allocation
Allocated to standard care (n=X)
Cycle 1, Day 1 (n=X)
Received allocated intervention (n=X)
Lost to follow up (n=X)
Withdrawn (n=X)
Cycle 2, Day 1, troponin, AEs (n=X)
Received allocated intervention (n=X)
Lost to follow up (n=X)
Withdrawn (n=X)
Cycle 3, Day 1, troponin, AEs (n=X)
Received allocated intervention (n=X)
Lost to follow up (n=X)
Withdrawn (n=X)
Cycle 4, Day 1, troponin, AEs (n=X)
Received allocated intervention (n=X)
Lost to follow up (n=X)
Withdrawn (n=X)
Cycle 5, Day 1, troponin, AEs (n=X)
Received allocated intervention (n=X)
Lost to follow up (n=X)
Withdrawn (n=X)
Cycle 6, Day 1, troponin, AEs (n=X)
Received allocated intervention (n=X)
Lost to follow up (n=X)
Withdrawn (n=X)
Cycle 1, Day 1 (n=X)
Received allocated intervention (n=X)
Lost to follow up (n=X)
Withdrawn (n=X)
Cycle 2, Day 1, troponin, AEs (n=X)
Received allocated intervention (n=X)
Lost to follow up (n=X)
Withdrawn (n=X)
Cycle 3, Day 1, troponin, AEs (n=X)
Received allocated intervention (n=X)
Lost to follow up (n=X)
Withdrawn (n=X)
Cycle 4, Day 1, troponin, AEs (n=X)
Received allocated intervention (n=X)
Lost to follow up (n=X)
Withdrawn (n=X)
Cycle 5, Day 1, troponin, AEs (n=X)
Received allocated intervention (n=X)
Lost to follow up (n=X)
Withdrawn (n=X)
Cycle 6, Day 1, troponin, AEs (n=X)
Received allocated intervention (n=X)
Lost to follow up (n=X)
Withdrawn (n=X)
End of Study, troponin, AEs echocardiogram, acceptability
Analysis: Completed follow up to EoS and were analysed (n=X), Excluded from analysis (give reasons) (n=X)

CONSORT 2010 checklist of information to include when reporting a randomised trial*

Section/Topic	Item No	Checklist item	Reported on page No
**Title and abstract**			
	1a	Identification as a randomised trial in the title	1
	1b	Structured summary of trial design, methods, results, and conclusions (for specific guidance see CONSORT for abstracts)	2
**Introduction**			
Background and objectives	2a	Scientific background and explanation of rationale	3,4,5
	2b	Specific objectives or hypotheses	5,7,8
**Methods**			
Trial design	3a	Description of trial design (such as parallel, factorial) including allocation ratio	5,6
	3b	Important changes to methods after trial commencement (such as eligibility criteria), with reasons	8,9
Participants	4a	Eligibility criteria for participants	5,6
	4b	Settings and locations where the data were collected	5
Interventions	5	The interventions for each group with sufficient details to allow replication, including how and when they were actually administered	6,7
Outcomes	6a	Completely defined pre-specified primary and secondary outcome measures, including how and when they were assessed	7,8
	6b	Any changes to trial outcomes after the trial commenced, with reasons	8,9
Sample size	7a	How sample size was determined	8,9
	7b	When applicable, explanation of any interim analyses and stopping guidelines	8,9
Randomisation:			
Sequence generation	8a	Method used to generate the random allocation sequence	6
	8b	Type of randomisation; details of any restriction (such as blocking and block size)	6
Allocation concealment mechanism	9	Mechanism used to implement the random allocation sequence (such as sequentially numbered containers), describing any steps taken to conceal the sequence until interventions were assigned	6,7
Implementation	10	Who generated the random allocation sequence, who enrolled participants, and who assigned participants to interventions	6
Blinding	11a	If done, who was blinded after assignment to interventions (for example, participants, care providers, those	7,9

For peer review only

		assessing outcomes) and how	
	11b	If relevant, description of the similarity of interventions	N/A
Statistical methods	12a	Statistical methods used to compare groups for primary and secondary outcomes	10
	12b	Methods for additional analyses, such as subgroup analyses and adjusted analyses	10
**Results**			
Participant flow (a diagram is strongly recommended)	13a	For each group, the numbers of participants who were randomly assigned, received intended treatment, and were analysed for the primary outcome	N/A
	13b	For each group, losses and exclusions after randomisation, together with reasons	N/A
Recruitment	14a	Dates defining the periods of recruitment and follow-up	N/A
	14b	Why the trial ended or was stopped	N/A
Baseline data	15	A table showing baseline demographic and clinical characteristics for each group	N/A
Numbers analysed	16	For each group, number of participants (denominator) included in each analysis and whether the analysis was by original assigned groups	N/A
Outcomes and estimation	17a	For each primary and secondary outcome, results for each group, and the estimated effect size and its precision (such as 95% confidence interval)	N/A
	17b	For binary outcomes, presentation of both absolute and relative effect sizes is recommended	N/A
Ancillary analyses	18	Results of any other analyses performed, including subgroup analyses and adjusted analyses, distinguishing pre-specified from exploratory	N/A
Harms	19	All important harms or unintended effects in each group (for specific guidance see CONSORT for harms)	N/A
**Discussion**			
Limitations	20	Trial limitations, addressing sources of potential bias, imprecision, and, if relevant, multiplicity of analyses	N/A
Generalisability	21	Generalisability (external validity, applicability) of the trial findings	N/A
Interpretation	22	Interpretation consistent with results, balancing benefits and harms, and considering other relevant evidence	N/A
**Other information**			
Registration	23	Registration number and name of trial registry	1,2
Protocol	24	Where the full trial protocol can be accessed, if available	N/A
Funding	25	Sources of funding and other support (such as supply of drugs), role of funders	11

*We strongly recommend reading this statement in conjunction with the CONSORT 2010 Explanation and Elaboration for important clarifications on all the items. If relevant, we also recommend reading CONSORT extensions for cluster randomised trials, non-inferiority and equivalence trials, non-pharmacological treatments, herbal interventions, and pragmatic trials. Additional extensions are forthcoming: for those and for up to date references relevant to this checklist, see www.consort-statement.org.

For peer review only - http://bmjopen.bmj.com/site/about/guidelines.xhtml

BMJ Open

Preventing Cardiotoxicity in Patients with Breast Cancer and Lymphoma: protocol for a multi-centre randomised controlled trial (PROACT)

Journal:	*BMJ Open*
Manuscript ID	bmjopen-2022-066252.R1
Article Type:	Protocol
Date Submitted by the Author:	18-Nov-2022
Complete List of Authors:	Maier, Rebecca; Newcastle University, Newcastle Clinical Trials Unit; Academic Cardiovascular Unit, South Tees, South Tees Hospitals NHS Foundation Trust<br>Plummer, Chris; Newcastle Upon Tyne Hospitals NHS Foundation Trust<br>Kasim, Adetayo; Durham University, Durham Research Methods Centre<br>Akhter, Nasima; Durham University, Department of Anthropology<br>Ogundimu, Emmanuel; University of Durham, Mathematical Sciences<br>Maddox, Jamie; Department of Haematology, The James Cook University Hospital, Marton Road, Middlesbrough United Kingdom TS4 3BW, Department of Haematology<br>Graham, Janine; South Tees Hospitals NHS Foundation Trust, Department of Oncology<br>Stewart, Michael; South Tees Hospitals NHS Foundation Trust, Department of Cardiology<br>Wardley, Andrew; Outreach Research & Innovation Group<br>Haney, Sophie; County Durham and Darlington NHS Foundation Trust<br>Vahabi, Sharareh; James Cook University Hospital, Cardiology<br>Oxenham, Helen; North Tees and Hartlepool NHS Foundation Trust<br>Humphreys, Alison; South Tees Hospitals NHS Foundation Trust, Department of Oncology<br>Cresti, Nicola; Newcastle Upon Tyne Hospitals NHS Foundation Trust, Department of Oncology<br>Verrill, Mark; Newcastle Upon Tyne Hospitals NHS Foundation Trust, Department of Oncology<br>Graham, Richard; South Tees Hospitals NHS Foundation Trust<br>Chang, Lisa; South Tees Hospitals NHS Foundation Trust, Academic Cardiovascular Unit, South Tees<br>Hancock, Helen; Newcastle University, Newcastle Clinical Trials Unit<br>Austin, David; James Cook University Hospital
**Primary Subject Heading**:	Cardiovascular medicine
Secondary Subject Heading:	Oncology
Keywords:	CHEMOTHERAPY, CARDIOLOGY, Breast tumours < ONCOLOGY, Lymphoma < ONCOLOGY, ONCOLOGY

Preventing Cardiotoxicity in Patients with Breast Cancer and Lymphoma: protocol for a multi-centre randomised controlled trial (PROACT)

Chief Investigator: **Dr David Austin**
Consultant Cardiologist
The James Cook University Hospital
South Tees Hospitals NHS Foundation Trust
Marton Road,
Middlesbrough, TS4 3BW
Tel: 01642 850850

Corresponding Author: Rebecca Maier
Affiliations: 1,2.
Email: rebecca.maier@newcastle.ac.uk

Clinical Trials Unit: **Newcastle Clinical Trials Unit**

Funder: National Institute of Health Research – Research for Patient Benefit Programme (project reference PB-PG-0815-20061)

Sponsor: South Tees Hospitals NHS Foundation Trust

Trial Identifiers: ClinicalTrials.gov: NCT03265574
EudraCT: 2017-001094-16
IRAS ID: 213348

Protocol Authors: Maier R 1,2, Plummer C 3, Kasim A 5, Akhter N 5, Ogundimu E 6, J Maddox 7, Graham J 7, Stewart M 8, Wardley A 9, Haney S 10, Vahabi S 8, Oxenham H 11, Humphreys A 7, Cresti N 3, Verrill M 3, Graham R 8, Chang L 2, Hancock H 1 and Austin D 2,8

1. Newcastle Clinical Trials Unit, Newcastle University
2. Academic Cardiovascular Unit, South Tees Hospitals NHS Foundation Trust
3. The Newcastle upon Tyne Hospitals NHS Foundation Trust
4. Centre for Research Methods, Durham University
5. Department of Anthropology, Durham University
6. Department of Mathematical Sciences, Durham University
7. Department of Oncology, South Tees Hospitals NHS Foundation Trust
8. Department of Cardiology, South Tees Hospitals NHS Foundation Trust
9. Outreach Research & Innovation Group
10. County Durham and Darlington NHS Foundation Trust
11. University Hospital of North Tees and Hartlepool

For peer review only - http://bmjopen.bmj.com/site/about/guidelines.xhtml

ABSTRACT – (276/300)

Introduction

Anthracyclines are included in chemotherapy regimens to treat several different types of cancer and are extremely effective. However, it is recognised that a significant side effect is cardiotoxicity; anthracyclines can cause irreversible damage to cardiac cells and ultimately impaired cardiac function and heart failure which may only be evident years after exposure. The PROACT trial will establish the effectiveness of the angiotensin-converting enzyme inhibitor (ACEI) enalapril maleate (enalapril) in preventing cardiotoxicity in patients with breast cancer and Non-Hodgkin Lymphoma (NHL) receiving anthracycline-based chemotherapy.

Methods and Analysis

PROACT is a prospective, randomised, open-label, blinded end-point, superiority trial which will recruit adult patients being treated for breast cancer and NHL at NHS hospitals throughout England. The trial aims to recruit 106 participants, who will be randomised to standard care (high dose anthracycline-based chemotherapy) plus enalapril (intervention), or standard care alone (control). Patients randomised to the intervention arm will receive enalapril (starting at 2.5 mg bd and titrating up to a maximum dose of 10mg bd), commencing treatment at least two days prior to starting chemotherapy and finishing three weeks after their last anthracycline dose. The primary outcome is the presence or absence of cardiac troponin T release at any time during anthracycline treatment, and one month after the last dose of anthracycline. Secondary outcomes will focus on cardiac function measured using echocardiogram assessment, adherence to enalapril, and side effects.

Ethics and Dissemination

A favourable opinion was given following Research Ethics Committee review by West Midlands - Edgbaston REC. Trial findings will be disseminated through engagement with patients, the oncology and cardiology communities, NHS management and commissioning groups, and through peer reviewed publication.

Trial Registration Number

ClinicalTrials.gov: NCT03265574

Strengths and Limitations of this Trial

1. Strong and ongoing patient and public involvement (PPI) and clinical consensus informed the robust randomised trial design, with 80% power.
2. Multi-centre UK trial with blinding of the primary outcome and the echo core laboratory reduces bias and will enable robust findings.

For peer review only - http://bmjopen.bmj.com/site/about/guidelines.xhtml

3. The intervention is low cost, and with decades of evidence indicating its safety; if findings from PROACT demonstrate effectiveness, its translation into patient benefit can be realised through rapid adoption into usual care.
4. Findings will have implications for multiple cancers across adult and paediatric settings where patients receive high dose anthracycline chemotherapy.
5. Two patient groups (Breast Cancer and Lymphoma) may make interpretation of results difficult if differences between the groups are found.

Introduction

Breast cancer is the most common malignancy among women worldwide, with over 55,000 new UK cases every year[1,2]. Lymphoma is the most common haematological malignancy and the sixth most common cancer in the UK, with Non-Hodgkin Lymphoma (NHL) affecting 14,000 patients per year[3]. Treatment for both conditions includes anthracycline chemotherapy (epirubicin or doxorubicin); however, anthracyclines can cause immediate, irreversible damage to cardiac cells and ultimately impaired cardiac function and heart failure which may only be evident years after exposure.[4] The long-term incidence is approximately 5% and may be higher in older patients.[5,6,7] Asymptomatic left ventricular systolic dysfunction (LVSD) is more common, evident in 9.7% of patients with breast cancer within 12 months.[8.] LVSD affects cardiac prognosis and limits subsequent cancer treatment options, particularly those that target the HER2 pathway such as trastuzumab (Herceptin).[8,9] In lymphoma, due to significant improvements in survival rates, there is also greater prevalence of anthracycline-induced cardiotoxicity in survivors.[10] Waiting until anthracycline-induced heart failure becomes clinically evident is ill-advised, with response to treatment poor and survival at 2 years of 40%; considerably worse than other causes of heart failure.[11]

The burden of cardiotoxicity is particularly important in both patient groups where 78% (breast) and 63% (NHL) will be alive 10 years following their cancer diagnosis.[1,12] Preventing cardiac damage would offer clear benefits to patients and substantial costs savings to the NHS.

Current Evidence Supporting the Rationale for the Trial

Anthracycline toxicity is thought to be due to the generation of reactive oxygen species causing cell death.[13,14] ACEI promote nitric oxide and inhibit the production of angiotensin II, which in turn reduces NAD(P)H oxidase responsible for superoxide formation. ACEI and angiotensin inhibition are protective of apoptosis in vascular endothelium and cardiac cells, prevent anthracycline cardiotoxicity in animal models and reduce oxidative stress.[15-19]

ACEI are of proven benefit in cardiovascular medicine, and are widely used, well tolerated and inexpensive (cost £2 per patient per month). Enalapril is the reference standard ACEI and has been extensively studied.[20, 21]

For peer review only - http://bmjopen.bmj.com/site/about/guidelines.xhtml

Treatment to prevent cardiotoxicity is attractive and feasible but is unproven. A Cochrane systematic review was unable to make definitive conclusions regarding the effectiveness of previously studied cardioprotective agents; no studies of ACEI were included.[22] A meta-review and further systematic review also found a lack of evidence to guide decision-making.[23,24] A European Society of Cardiology (ESC) position paper and the recently published ESC guideline on cardio-oncology also draw the same conclusions on preventative therapy.[25,26]

An Italian study assessed enalapril treatment in patients with a positive troponin during chemotherapy (not all were anthracycline-based regimens). By the second month, 41% of patients in the control group (n=58) had persistent troponin elevation, compared to 4% in the enalapril group (n=56). Based on echocardiography, no cardiotoxicity in the enalapril group was observed; cardiotoxicity was identified in 43% of controls ($p<0.05$).[27]

Small European studies assessed preventative strategies in lower doses of anthracyclines (average 240 mg/m^2), but demonstrated that LVEF measured by cardiac MRI declined less in patients treated with candestartan[28]; similar results are seen in those treated with enalapril and carvedilol.[29] Subsequent 2 year follow up of the PRADA study suggested only a small decline in LVEF in patients receiving lower dose anthracyclines, and no significant between group differences.[30]

ICOS ONE randomised patients to enalapril or "troponin-triggered" enalapril and found no difference between the two strategies.[31] Median dose of anthracyclines was low (180mg/m^2) and the most enalapril was typically prescribed at 2.5 mg bd.[31] The rate of cardiotoxicity at 3 years was low.[31,32]

A UK based clinical trial, CARDIAC CARE (ISRCTN24439460), is testing a different hypothesis to PROACT; patients with breast cancer or NHL will have troponin-guided randomisation to treatment with beta-blockers and ACEI or usual care. CARDIAC CARE is currently recruiting and will provide complimentary information to PROACT on its completion.[33]

Troponin T is a sensitive marker of early cardiac cell death and as such there is the potential for troponin release to be "turned off" if enalapril is effective. Audit data from 36 patients and 143 samples, showed a positive troponin T in 47% of patients who received >300 mg/m^2 of anthracycline in a six-cycle regimen. Troponin T was chosen as the primary outcome measure on this basis, and was endorsed by our patient groups. It is known from previous research that troponin correlates with subsequent changes in LV function, and importantly that a negative troponin during and at one month post chemotherapy essentially excludes significant cardiotoxicity.[34,35]

There are currently no definitive trials of ACEI in the prevention of anthracycline cardiotoxicity in patients receiving the highest contemporary doses of chemotherapy. PROACT will determine the effectiveness of enalapril in preventing cardiotoxicity in patients receiving high-dose anthracycline-based chemotherapy for breast cancer and NHL. Results will also inform practice for other cancer types. Findings will directly inform clinical practice in confirming whether enalapril should be given routinely to

For peer review only - http://bmjopen.bmj.com/site/about/guidelines.xhtml

patients receiving anthracycline-based chemotherapy for breast cancer and NHL.

Increasing breast cancer and NHL survival, the frequency and impact of cardiotoxicity and the potential for a simple, safe and cheap preventative treatment makes PROACT highly important for patients and the NHS.

METHODS AND ANALYSIS

Study Design

PROACT is a prospective, randomised, open-label, blinded end-point, superiority trial in patients undergoing anthracycline-based chemotherapy for breast cancer or NHL. Patients will be randomised to receive enalapril (intervention) or control (standard care).

The trial will answer the question 'Can troponin release be prevented by enalapril in patients undergoing high dose anthracycline chemotherapy treatment for cancer?

Setting

Patients due to receive high dose anthracycline-based chemotherapy for their breast cancer or NHL at participating NHS Trusts will be offered recruitment to the trial. All sites can accommodate the needs of this trial including research nurse support, facilities for trial interventions and assessments, and British Society of Echocardiography (BSE) accredited echocardiographers, advanced trainee, or consultant cardiologists, to carry out echocardiograms in accordance with the trial protocol. The trial began recruiting in September 2017, and is due to report in late 2023.

Eligibility Criteria

Inclusion criteria:

- Adult patients with histopathologically* confirmed breast carcinoma who have received surgery for their breast cancer;
- Planned to receive 6 cycles of **EC 90** (total planned dose 540 mg/m^2 epirubicin) or **FEC 75** (total planned dose 450 mg/m^2 epirubicin) adjuvant chemotherapy regimen;

OR

- Adult patients with histopathologically confirmed non-Hodgkin lymphoma planned to receive 6 cycles of **R-CHOP or CHOP** (total planned dose 300mg/m^2 doxorubicin) chemotherapy**

AND

- Written informed consent.

**Patients with HER2+ breast cancer are eligible for inclusion.*

***Patients who will receive an alternative anti-CD20 monoclonal antibody are eligible (for example O-CHOP), as long as the total planned doxorubicin dose is ≥300mg/m^2 over 6 cycles*

For peer review only - http://bmjopen.bmj.com/site/about/guidelines.xhtml

Exclusion criteria:

- positive baseline cardiac troponin T (≥14 ng/L);
- known contraindication to ACE inhibitor e.g. renal artery stenosis, severe aortic stenosis;
- are taking, or have a previous intolerance to ACEI (e.g. angioedema);
- patient already taking other agents acting on the renin-angiotensin-aldosterone system e.g., Aliskiren, angiotensin receptor blockers (ARBs), Entresto (sacubitril/valsartan), spironolactone, eplerenone;
- LVEF <50%;
- estimated GFR < 30 mL/min/1.73m^2 at baseline;
- hyperkalaemia defined as serum potassium ≥5.5mmol/L;
- symptomatic hypotension, or Systolic Blood Pressure <100mmHg;
- poorly-controlled hypertension (Blood Pressure >160/100mmHg, or ambulatory BP of 150/95mmHg);
- previous myocardial infarction;
- known metastatic breast cancer;
- previous exposure to anthracycline chemotherapy;
- are pregnant or breastfeeding;
- previous trastuzumab treatment or planned trastuzumab treatment within four weeks following anthracycline chemotherapy;
- for patients of childbearing potential: refusal to use adequate contraception throughout trial;***
- any other invasive cancer diagnosed and treated in the past 5 years;
- symptomatic or severe asymptomatic radiation-induced cardiac disease;
- participation in other interventional medicinal trials in the past 6 months;
- judgement by the investigator that the patient has a prognosis of < 1 year or are unlikely to complete 6 cycles of chemotherapy.
- judgement by the investigator that the patient is high risk for tumour lysis syndrome (applicable only to NHL patients).
- judgement by the Investigator that the patient should not participate in the study, (e.g., if patient is unlikely to comply with study procedures, restrictions, and requirements.

***Female patients between the ages of 18 and 50 will receive a pregnancy test at baseline.

Randomisation

Randomisation will use a minimisation scheme which adjusts for baseline factors; the minimisation scheme will account for the planned 6 cycle chemotherapy regimen (EC 90 or FEC 75, or R-CHOP), and, in breast cancer HER2 status (positive or negative). Patients will be randomised 1:1 to either standard care plus enalapril, or standard care only by members of the research team at each centre using a 24-hour, central, secure, web-based randomisation system with concealed allocation (procured from Sealed Envelope Ltd).

For peer review only - http://bmjopen.bmj.com/site/about/guidelines.xhtml

Trial Intervention

Standard Care

All patients in both arms will be planned to receive 6 cycles of chemotherapy as part of standard care. There is no placebo in the standard care alone arm.

The following regimens are permitted within the trial:

Breast Cancer regimens:

- EC 90; Epirubicin 90mg/m^2, Cyclophosphamide 600mg/m^2
- FEC 75; Fluorouracil 600mg/m^2, Epirubicin 75mg/m^2, Cyclophosphamide 600mg/m^2

Non Hodgkin Lymphoma regimens:

- R-CHOP* or CHOP; (Rituximab 375mg/m^2), Cyclophosphamide 750mg/m^2, Doxorubicin 50mg/m^2, Vincristine 1.4mg/m^2 (max 2mg), Prednisolone 40mg/m^2 (for 5 days).

**Patients who receive an alternative anti-CD20 monoclonal antibody are eligible (for example O-CHOP), as long as the total planned doxorubicin dose is ≥300mg/m^2 over 6 cycles*

Intervention arm

Patients randomised to the intervention arm will receive enalapril in addition to standard care. Patients will commence on a 2.5mg bd dose at least two days prior to their first dose of anthracycline chemotherapy, with the aim to up-titrate the dose to 5mg bd, then 10mg bd over a maximum of three dose evaluation assessments prior to cycle 2. Chemotherapy will not be delayed by taking part in the trial. Some clinicians/centres routinely withhold ACEI on the morning of rituximab therapy; this is allowed per protocol.

Dose evaluation assessment visits will take place between two and seven days after the start of each dose level. If the patient has systolic BP ≥100 mmHg, normal serum potassium (potassium <5.5 mmol/L), and stable renal function (serum creatinine <30 μmol/L) the dose will be increased to the next dose level. If clinical opinion is that the patient is unlikely to tolerate the higher dose, the dose will remain the same. If the patient has a ≥30 μmol/L increase in serum creatinine levels since the last assessment compared to baseline, new hyperkalaemia (potassium ≥5.5mmol/L), or symptomatic hypotension (SBP <100mmHg) then enalapril will be permanently discontinued. Patients will remain on the maximum enalapril dose reached for the duration of their chemotherapy, and until three weeks following their last dose of anthracycline. Temporary halts of up to 14 days will be allowed, with re-introduction and dose reductions at the discretion of the Investigator. Extensive PPI work at the point of trial design, and financial cost, led to a decision not to include a placebo in the control arm.

Blinding

PROACT is an open label trial. The trial employs a prospective randomised blinded endpoint design; analysis of troponin T and troponin I will be completed by laboratory staff who are blinded to the patients' trial allocation, as detailed in the trial PROACT laboratory manual. The trial management team,

For peer review only - http://bmjopen.bmj.com/site/about/guidelines.xhtml

statistics, and clinical teams will remain blinded to the troponin results until the end of the trial. The Data Manager, and an un-blind monitor separate to the trial team, will have access to the troponin results for the purposes of monitoring and data cleaning.

All echocardiograms will be sent to an independent Core Laboratory for assessment by a BSE accredited echocardiographer/or an advanced trainee or consultant cardiologist blind to the intervention.

Outcomes

Primary outcome:

The primary outcome is cardiotoxicity measured as presence (≥14ng/L) of cardiac troponin T release at any time during anthracycline treatment, and one month after the last dose of anthracycline.

Secondary outcomes:

- Cardiac function will be assessed by echocardiogram, including global longitudinal strain (GLS), and measurements of left ventricular ejection fraction (LVEF), at baseline and following completion of chemotherapy;
- Cardiotoxicity will be measured as cardiac troponin I release during chemotherapy and at one month after the last dose of anthracycline;
- Adherence to enalapril will be measured according to patient diaries throughout the trial;
- All adverse events, and adverse reactions including those that are serious and unexpected will be recorded from the day of randomisation until the last visit or until withdrawal; adverse events considered related to enalapril will be followed until resolution, a stable outcome or death.
- Anxiety or distress related to trial participation will be measured at the last study visit;
- Cancer and chemotherapy outcomes will be characterised in the population.

Protocol Changes

Breast cancer management

Shortly after the trial opened to recruitment, changes in the clinical management of patients with breast cancer reduced the number of patients potentially eligible patients. The first was the widespread adoption of genomic testing to calculate the Oncotype Dx recurrence score, which reduced the number of patients recommended for adjuvant chemotherapy by approximately 30% in our recruiting hospitals.
In addition, in July 2018 NICE published a clinical guideline on the management of breast cancer which included recommending a taxane to be routinely offered alongside an anthracycline in adjuvant chemotherapy (FEC-T chemotherapy); in the development of PROACT, we identified patients receiving FEC-T as being at low risk of cardiotoxicity. It was decided to include NHL patients in the trial to increase the number of potentially eligible patients.

COVID 19 impact

For peer review only - http://bmjopen.bmj.com/site/about/guidelines.xhtml

PROACT clinical trial delivery has been significantly affected by the COVID 19 pandemic. The trial was paused to recruitment in March 2020, and whilst it has re-opened to recruitment, ongoing impact on non-COVID research in the NHS, and in particular recruitment to Oncology clinical trials, is well documented. NIHR have agreed two funded extensions to enable the study to complete.

Sample Size

In light of the unprecedented challenges, the trial team, in agreement with funder, Trial Steering Committee (TSC) and Independent Data Monitoring and Ethics Committee (IDMEC) agreed to recalculate the sample size by changing from 90% to 80% power, using the same assumptions as in the original sample size calculation.

Assuming alpha of 5%, and 80% power, 106 patients are needed to detect a reduction in the proportion of patients with cardiac troponin T present from 47% to 20% using a two-sided Fisher's exact test; additional recruitment to account for attrition is planned. Observational data was used to provide us with the estimate of troponin T elevation in the standard care arm; there are other potential causes of an elevated troponin, such as infection, during chemotherapy and clinical consensus indicated that a rate of 20% in elevated troponin would fully account these. There is also a consensus within the clinical community that, a large effect size will be necessary to convince the clinical community to change the pathway of care for these patients. The original sample size at 90% power was 140 patients, inflated to 170 patients to account for attrition.

Trial Procedures

Patients due to undergo 6 cycles of anthracycline based chemotherapy for breast cancer or NHL at participating centres are identified by the clinical research team and approached about the trial, including given an information sheet and consent form. After discussion, consent is sought, baseline assessments performed and eligibility checked and confirmed. Eligible patients are randomised, and their General Practitioners informed. A full schedule of events is detailed in Table 1, and a participant flow chart provided in Figure 1. Patients have assessments at baseline, dose evaluation visits (up to three for those receiving the intervention) within 72 hours prior to day 1 of each chemotherapy cycle, and at trial completion (4 weeks following the last dose of anthracycline). A separately funded study is allowing patients to be followed for one year, and all patients are asked to consent to longer term follow up.

Baseline activities include consent, medical history, height/weight, NYHA class, BP check, bloods, eGFR and eligibility check.

For peer review only - http://bmjopen.bmj.com/site/about/guidelines.xhtml

Table 1: PROACT Schedule of Events

	Baseline (up to 6 weeks prior to randomisation)	Day 1 (at least 2 days prior to Cycle 1 Day 1)	Dose evaluation visit 1	Dose evaluation visit 2	Dose evaluation visit 3	Cycle 1, Day 1	Cycle 2, Day 1	Cycle 3, Day 1	Cycle 4, Day 1	Cycle 5, Day 1	Cycle 6, Day 1	Trial Completion Visit	1-year Follow-up Visit
**Consent**	X												
**Demographics**	X												
**Medical History**	X												X
**Cancer history**	X												X
**Performance Status**	X												X
**NYHA Class**	X												X
**Concomitant medications**	X	X	X	X	X	X	X	X	X	X	X	X	X
**Physical Assessment (height and weight)**	X												X
**Blood Pressure**	X		X	X	X	X	X	X	X	X	X	X	X
**Troponin T (baseline sample)**	X												
**Troponin T (post-baseline)**							X	X	X	X	X	X	
**Troponin I**	X						X	X	X	X	X	X	
**U+Es**	X		X	X	X		X	X	X	X	X	X	
**eGFR**	X												
**Pregnancy test**	X												
**Echocardiogram**	X											X	X
**Eligibility check**	X												
**Randomisation**	X												
**Enalapril (intervention group only)**		X	X	X	X	X	X	X	X	X	X		
**Adherence to enalapril (intervention group only)**		X	X	X	X	X	X	X	X	X	X	X	
**Standard care chemotherapy (all patients)**						X	X	X	X	X	X		
**Adverse Events**		X	X	X	X	X	X	X	X	X	X	X	
**Buccal swab for future research**	X												
**Blood sample for future research**	X							X		X		X	
**Acceptability of trial interventions assessment**									X			X	

For peer review only - http://bmjopen.bmj.com/site/about/guidelines.xhtml

Troponin T and Troponin I

Blood sampling for troponin T and I will be performed at baseline, within 72 hours prior to chemotherapy (Cycle 2 onwards), and trial completion (30 days after the final anthracycline dose). Troponin T samples will provide the primary outcome data. Samples will be processed and sent to a core laboratory for central analysis; the central laboratory will be blind to treatment allocation.

Echocardiogram

Cardiac function is assessed via transthoracic echocardiography (TTE) at baseline, four weeks after the last anthracycline dose and one year after the date of final chemotherapy. All echocardiograms will be assessed by a Core Laboratory who will be blind to participant treatment. Local reporting of TTEs will be as per local hospital practice; the data reported by the Core Laboratory (including GLS and LVEF) will be analysed as part of the secondary outcomes for the trial.

Trial Anxiety or Distress Participation Assessment

At the start of cycle 4 and at the end of study visit (1 month after the last chemotherapy treatment), patients will be asked to complete a short questionnaire to understand if taking part in the trial has resulted in any anxiety or distress. This outcome was requested by the funding panel and will help to understand the impact on patients of a change to standard care in the event that PROACT demonstrates the effectiveness of enalapril in this setting.

Statistical Analysis

Data cleaning and analysis will be provided by staff within NCTU and Durham University. Primary analysis will follow intention to treat principles with patient data analysed according to randomisation and irrespective of intervention received; other analysis groups such as per-protocol may be considered subsequently. Every effort will be made to retain and include all patients who are part of the trial.

A full statistical analysis plan (SAP) will be developed for the outcome measures and agreed with the Independent Data Monitoring and Ethics Committee (IDMEC) and Chief Investigator prior to any analysis being undertaken.

Outcome data will be analysed at the end of the main study, no interim analysis is planned. Follow-up data will be analysed and reported separately.

The primary analysis of presence or absence of cardiac troponin T will be assessed using logistic regression and accounting for minimisation factors. Analysis of the secondary endpoints will be dependent upon the nature of the specific endpoint and data structure; global longitudinal strain (GLS) and left ventricular ejection fraction (LVEF) will be analysed as a change from baseline using regression model to compare intervention and control groups.

Cardiac troponin I data will also be analysed using logistic regression and account for minimisation factors. Changes in cardiac troponin T and I will also be analysed as a continuous variable. Additional analysis of the primary endpoint will be performed using logistic regression to account for baseline

For peer review only - http://bmjopen.bmj.com/site/about/guidelines.xhtml

factors (regimen and HER2 status). Adverse events data will be analysed using cross-tabulation. Sensitivity analysis will also be performed for adherence to the protocol and compare breast cancer and non-breast cancer patients.

Trial Conduct and governance

The Trial Management Group is responsible for the day to day management of the trial, overseeing all aspects to ensure that the protocol is adhered to and taking appropriate actions to ensure patient safety and data integrity. The IDMEC review trial outcomes (including adverse events and serious adverse events), provide advice on the ongoing conduct and safety of the trial and report recommendations to the TSC. The TSC, where independent members are the majority (including patient representatives), provides overall supervision, monitors progress and conduct and advises on the trial. The IDMEC and TSC will meet every six months.

Patient and Public Involvement

Initial trial ideas were discussed with patients who had recently finished chemotherapy treatment for breast cancer the Maggie's Centre in Newcastle in 2015 and at a breast cancer support group in North Yorkshire and again with patients from the Maggie's Centre; trial ideas were developed between each discussion based on their feedback prior to submission of the application.

The trial also has two patient representatives who sit on the Trial Steering Committee; one patient previously treated for breast cancer, the other previously treated for NHL. Neither of these patients are trial participants, nor are they employed by any organisation directly involved in the trial conduct.

Ethics and dissemination

All clinical research raises ethical issues, and the trial team carefully thought through potential issues and aimed to address these in the trial design. This study does not include patients who will lack capacity, nor minors. This trial is not based in an emergency setting. The trial is conducted in accordance with the protocol (current version 8.0, dated 3rd March 2022), Good Clinical Practice, the favourable ethical opinion (from West Midlands - Edgbaston REC) and the MHRA notice of no-objection. All patients provide written informed consent prior to participation. Enalapril has been widely used for over thirty years, is well tolerated without significant side effects.

If this research demonstrates that enalapril is effective in preventing heart damage in patients receiving anthracycline chemotherapy this will have a significant impact not just for patients with breast cancer and NHL, but for both adult and paediatric patients with other types of cancer treated with chemotherapy regimens containing anthracyclines. In addition to reduced morbidity and mortality for patients, enalapril will provide significant savings for the NHS.

We will publish the findings in peer-reviewed journals, and disseminate results to patients, and the international clinical community.

For peer review only - http://bmjopen.bmj.com/site/about/guidelines.xhtml

Acknowledgements

We are grateful to members of the IDMEC, and TSC for their support of PROACT. We would like to acknowledge the considerable work being undertaken by Principal Investigators and members of the site teams in support of this trial, and the PROACT trial team at Newcastle Clinical Trials Unit.

Authors Contributions

DA and CJP conceived the idea for the trial. DA, HCH, RHM, ASK, CJP, MV, NC, JG, JM, MS, AW, SH, HO, AH, RG co-designed the trial, secured funding from the NIHR RfPB and wrote the full trial protocol with substantial input from LC. DA is the Chief Investigator; RHM and HCH provide methodological input and oversee NCTU activity. EO leads the statistical aspects and analysis, overseeing NA. SV undertakes echocardiogram review and reporting at the core echocardiogram laboratory. This paper was drafted from the approved version of the protocol; all authors commented and amended drafts of the paper and approved the final manuscript.

Funding Statement

This work is supported by the National Institute for Health Research (NIHR) Research for Patient Benefit (RfPB) Programme (Ref: PB-PG-0815-20061).

The PROACT Trial is sponsored by South Tees Hospitals NHS Foundation Trust.

Conflicts of Interest

Professor Adetayo Kasim's contribution was during his employment by Durham University. He currently works for UCB Biopharma, UK.

Professor Andrew Wardley currently works for Outreach Research & Innovation Group, and was employed by The Christie NHS Foundation Trust, Manchester when the grant was awarded.

Dr David Austin has previously received speaker fees from Astra Zeneca, Pfizer and Philips/Volcano. None were directly relevant to PROACT

David Austin https://orcid.org/0000-0003-4606-1055

Rebecca Maier: https://orcid.org/0000-0002-7350-3288

Helen Hancock: https://orcid.org/0000-0002-1494-8551

Adetayo Kasim: https://orcid.org/0000-0002-0411-3059

Nicola Cresti: https://orcid.org/0000-0002-0495-4472

Jamie Maddox: https://orcid.org/0000-0002-7995-7934

Nasima Akhter: https://orcid.org/0000-0002-5424-1593

Emmanuel Ogundimu: https://orcid.org/0000-0001-9252-9275

Andrew Wardley: https://orcid.org/0000-0002-9639-0888

Licence Statement

I, the Submitting Author has the right to grant and does grant on behalf of all authors of the Work (as defined in the below author licence), an exclusive licence and/or a non-exclusive licence for contributions

For peer review only - http://bmjopen.bmj.com/site/about/guidelines.xhtml

from authors who are: i) UK Crown employees; ii) where BMJ has agreed a CC-BY licence shall apply, and/or iii) in accordance with the terms applicable for US Federal Government officers or employees acting as part of their official duties; on a worldwide, perpetual, irrevocable, royalty-free basis to BMJ Publishing Group Ltd ("BMJ") its licensees and where the relevant Journal is co-owned by BMJ to the co-owners of the Journal, to publish the Work in BMJ Open and any other BMJ products and to exploit all rights, as set out in our licence.

The Submitting Author accepts and understands that any supply made under these terms is made by BMJ to the Submitting Author unless you are acting as an employee on behalf of your employer or a postgraduate student of an affiliated institution which is paying any applicable article publishing charge ("APC") for Open Access articles. Where the Submitting Author wishes to make the Work available on an Open Access basis (and intends to pay the relevant APC), the terms of reuse of such Open Access shall be governed by a Creative Commons licence – details of these licences and which Creative Commons licence will apply to this Work are set out in our licence referred to above.

REFERENCES

1. Cancer Research UK. Breast cancer statistics.http://www.cancerresearchuk.org/health-professional/cancer-statistics/statistics-by-cancer-type/breast-cancer. Accessed 26th April 2022.
2. World Cancer Research Fund International. Breast cancer statistics. https://www.wcrf.org/cancer-trends/breast-cancer-statistics/ Accessed 26th April 2022.
3. Cancer Research UK Non-Hodgkin Lymphoma Statistics https://www.cancerresearchuk.org/health-professional/cancer-statistics/statistics-by-cancer-type/non-hodgkin-lymphoma Accessed 26th April 2022.
4. Groarke JD, Nohria A. Anthracycline Cardiotoxicity: A New Paradigm for an Old Classic. Circulation 2015;**131**(22):1946-9.
5. Swain SM, Whaley FS, Ewer MS. Congestive heart failure in patients treated with doxorubicin: a retrospective analysis of three trials. Cancer 2003;**97**(11):2869-79.
6. Pinder MC, Duan Z, Goodwin JS*, et al.* Congestive heart failure in older women treated with adjuvant anthracycline chemotherapy for breast cancer. J Clin Oncol 2007;**25**(25):3808-15.
7. Steinherz LJ, Steinherz PG, Tan CC*, et al.* Cardiac toxicity 4 to 20 years after completing anthracycline therapy. JAMA 1991;**266**(12):1672-77.
8. Cardinale D, Colombo A, Bacchiani G*, et al.* Early Detection of Anthracycline Cardiotoxicity and Improvement With Heart Failure Therapy. Circulation 2015;**131**(22):1981-8.
9. Piccart-Gebhart MJ, Procter M, Leyland-Jones B*, et al.* Trastuzumab after Adjuvant Chemotherapy in HER2-Positive Breast Cancer. N Engl J Med 2005;**353**(16):1659-72.
10. Ewer M, Ewer S. Cardiotoxicity of anticancer treatments. Nature Reviews Cardiology. 2015;**12**(9):547-558.
11. Felker GM, Thompson RE, Hare JM*, et al.* Underlying Causes and Long-Term Survival in Patients with Initially Unexplained Cardiomyopathy. N Engl J Med 2000;**342**(15):1077-84.

For peer review only - http://bmjopen.bmj.com/site/about/guidelines.xhtml

12. Quaresma M, Coleman MP, Rachet B. 40-year trends in an index of survival for all cancers combined and survival adjusted for age and sex for each cancer in England and Wales, 1971-2011: a population-based study. Lancet 2015;**385**(9974):1206-18.
13. Münzel T, Keaney JF. Are ACE Inhibitors a “Magic Bullet” Against Oxidative Stress? Circulation 2001;**104**(13):1571-74.
14. Rajagopalan S, Politi PM, Sinha BK*, et al.* Adriamycin-induced free radical formation in the perfused rat heart: implications for cardiotoxicity. Cancer Res 1988;**48**(17):4766-9.
15. Vaynblat M, Shah HR, Bhaskaran D*, et al.* Simultaneous angiotensin converting enzyme inhibition moderates ventricular dysfunction caused by doxorubicin. Eur J Heart Fail 2002;**4**(5):583-6.
16. Sacco G, Mario B, Lopez G*, et al.* ACE inhibition and protection from doxorubicin-induced cardiotoxicity in the rat. Vascul Pharmacol 2009;**50**(5-6):166-70.
17. Noda K, Kobara M, Hamada J*, et al.* Additive amelioration of oxidative stress and cardiac function by combined mineralocorticoid and angiotensin receptor blockers in postinfarct failing hearts. J Cardiovasc Pharmacol 2012;**60**(2):140-9.
18. Ferrari R, Fox K. Insight into the Mode of Action of ACE Inhibition in Coronary Artery Disease. Drugs 2009;**69**(3):265-77.
19. Galvez AS, Fiedler JL, Ocaranza MP*, et al.* Perindopril regulates beta-agonist-induced cardiac apoptosis. J Cardiovasc Pharmacol 2005;**46**(3):255-61.
20. McMurray JJV, Packer M, Desai AS*, et al.* Angiotensin–Neprilysin Inhibition versus Enalapril in Heart Failure. N Engl J Med 2014;**371**(11):993-1004.
21. British National Formulary. https://www.evidence.nhs.uk/formulary/bnf/current Accessed 28th November 2015.
22. van Dalen EC, Caron HN, Dickinson HO*, et al.* Cardioprotective interventions for cancer patients receiving anthracyclines. Cochrane Database Syst Rev 2011(6):CD003917.
23. Kalam K, Marwick TH. Role of cardioprotective therapy for prevention of cardiotoxicity with chemotherapy: a systematic review and meta-analysis. Eur J Cancer 2013;**49**(13):2900-9.
24. Conway A, McCarthy AL, Lawrence P*, et al.* The prevention, detection and management of cancer treatment-induced cardiotoxicity: a meta-review. BMC Cancer 2015;**15**(1):366.
25. Zamorano JL, Lancellotti P, Rodriguez Muñoz D*, et al.* 2016 ESC Position Paper on cancer treatments and cardiovascular toxicity developed under the auspices of the ESC Committee for Practice Guidelines. The Task Force for cancer treatments and cardiovascular toxicity of the European Society of Cardiology (ESC) 2016;**37**(36):2768-2801
26. Lyon AR, López-Fernández T, Couch LS, et al., 2022 ESC Guidelines on cardio-oncology developed in collaboration with the European Hematology Association (EHA), the European Society for Therapeutic Radiology and Oncology (ESTRO) and the International Cardio-Oncology Society (IC-OS): Developed by the task force on cardio-oncology of the European Society of Cardiology (ESC), European Heart Journal, Volume 43, Issue 41, 1 November 2022, Pages 4229–4361, https://doi.org/10.1093/eurheartj/ehac244
27. Cardinale D, Sandri MT, Colombo A*, et al.* Prognostic value of troponin I in cardiac risk stratification of cancer patients undergoing high-dose chemotherapy. Circulation

For peer review only - http://bmjopen.bmj.com/site/about/guidelines.xhtml

2004;**109**(22):2749-54

28. Gulati G, Heck SL, Ree AH*, et al.* Prevention of cardiac dysfunction during adjuvant breast cancer therapy (PRADA): a 2 × 2 factorial, randomized, placebo-controlled, double-blind clinical trial of candesartan and metoprolol. European Heart Journal 2016;**37**(21):1671-80
29. Bosch X, Rovira M, Sitges M*, et al.* Enalapril and carvedilol for preventing chemotherapy-induced left ventricular systolic dysfunction in patients with malignant hemopathies: the OVERCOME trial (preventiOn of left Ventricular dysfunction with Enalapril and caRvedilol in patients submitted to intensive ChemOtherapy for the treatment of Malignant hEmopathies). J Am Coll Cardiol 2013;**61**(23):2355-62.
30. Lagethon Heck S, Hansen Rec A, Mecinaj A, *et al.* Prevention of cardiac dysfunction during adjuvant breast cancer therapy (PRADA): Extended follow up of a 2x2 factorial, randomised, placebo-controlled, double blind trial of candesartan and metoprolol. Circulation 2021; **143** (25): 2431-2440
31. Cardinale D, Ciceri F, Latini R, *et al*. Anthracycline-induced cardiotoxicity: A multicenter randomised trial comparing two strategies for guiding prevention with enalapril: The International CardioOncology Society-one trial. Eur J Cancer 2018 **94**:126-137.
32. Meessen JMTA, Cardinale D, Ciceri F, *et al*. Circulating biomarkers and cardiac function over 3 years after chemotherapy with anthracyclines: the ICOS-ONE trial. ESC Heart Fail. 2020; **7**(4):1452-1466.
33. CARDIAC CARE The Cardiac Care Trial. http://www.isrctn.com/ISRCTN24439460 accessed 26th April 2022.
34. Cardinale D, Sandri MT, Martinoni A*, et al.* Left ventricular dysfunction predicted by early troponin I release after high-dose chemotherapy. J Am Coll Cardiol 2000;**36**(2):517
35. Cardinale D, Sandri MT, Colombo A*, et al.* Prognostic value of troponin I in cardiac risk stratification of cancer patients undergoing high-dose chemotherapy. Circulation 2004;**109**(22):2749-54.

Figure 1: PROACT Trial Flow Diagram

For peer review only

For peer review only - http://bmjopen.bmj.com/site/about/guidelines.xhtml

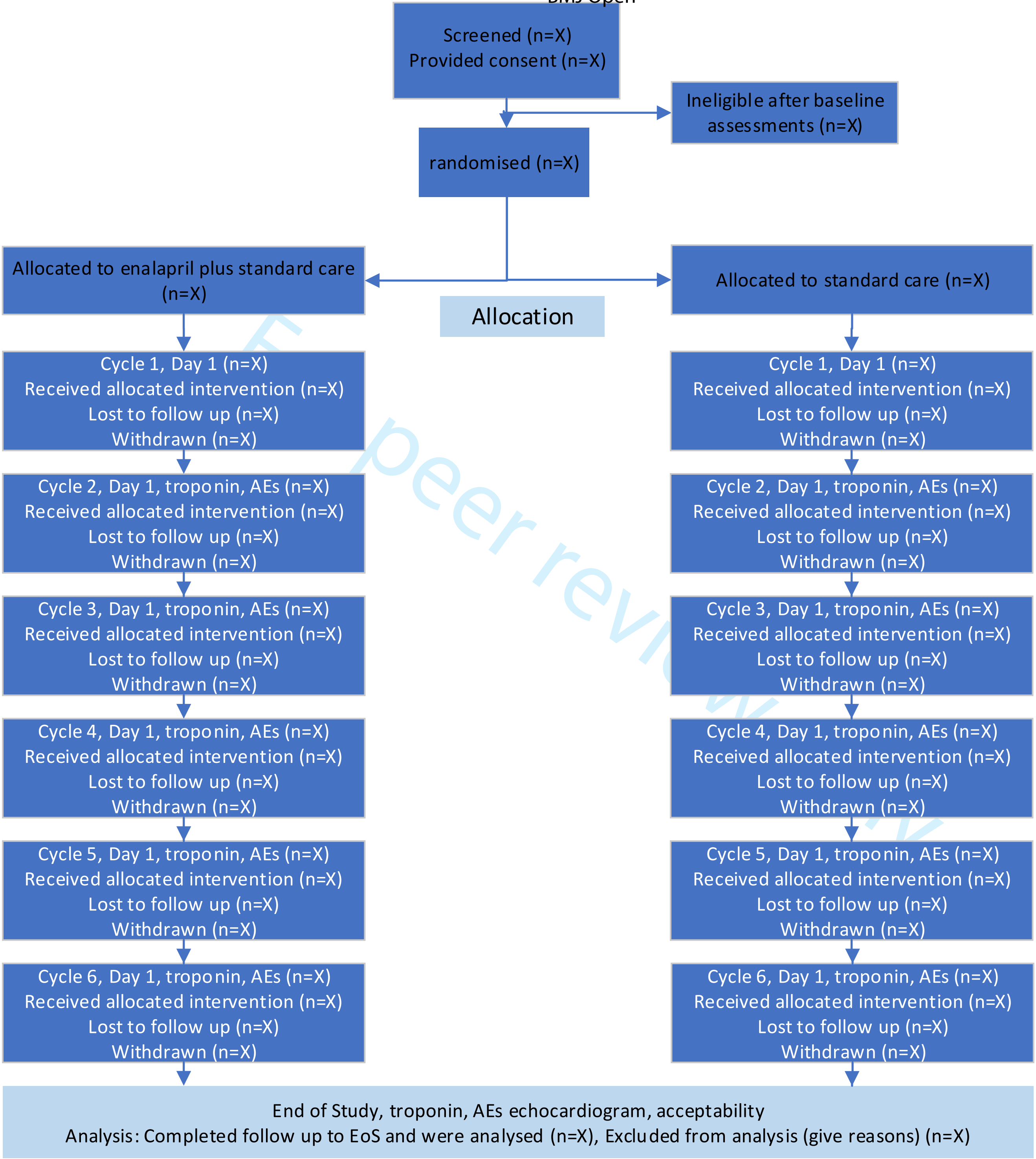
Screened (n=X)
Provided consent (n=X)
Ineligible after baseline assessments (n=X)
randomised (n=X)
Allocated to enalapril plus standard care (n=X)
Allocation
Allocated to standard care (n=X)
Cycle 1, Day 1 (n=X)
Received allocated intervention (n=X)
Lost to follow up (n=X)
Withdrawn (n=X)
Cycle 2, Day 1, troponin, AEs (n=X)
Received allocated intervention (n=X)
Lost to follow up (n=X)
Withdrawn (n=X)
Cycle 3, Day 1, troponin, AEs (n=X)
Received allocated intervention (n=X)
Lost to follow up (n=X)
Withdrawn (n=X)
Cycle 4, Day 1, troponin, AEs (n=X)
Received allocated intervention (n=X)
Lost to follow up (n=X)
Withdrawn (n=X)
Cycle 5, Day 1, troponin, AEs (n=X)
Received allocated intervention (n=X)
Lost to follow up (n=X)
Withdrawn (n=X)
Cycle 6, Day 1, troponin, AEs (n=X)
Received allocated intervention (n=X)
Lost to follow up (n=X)
Withdrawn (n=X)
Cycle 1, Day 1 (n=X)
Received allocated intervention (n=X)
Lost to follow up (n=X)
Withdrawn (n=X)
Cycle 2, Day 1, troponin, AEs (n=X)
Received allocated intervention (n=X)
Lost to follow up (n=X)
Withdrawn (n=X)
Cycle 3, Day 1, troponin, AEs (n=X)
Received allocated intervention (n=X)
Lost to follow up (n=X)
Withdrawn (n=X)
Cycle 4, Day 1, troponin, AEs (n=X)
Received allocated intervention (n=X)
Lost to follow up (n=X)
Withdrawn (n=X)
Cycle 5, Day 1, troponin, AEs (n=X)
Received allocated intervention (n=X)
Lost to follow up (n=X)
Withdrawn (n=X)
Cycle 6, Day 1, troponin, AEs (n=X)
Received allocated intervention (n=X)
Lost to follow up (n=X)
Withdrawn (n=X)
End of Study, troponin, AEs echocardiogram, acceptability
Analysis: Completed follow up to EoS and were analysed (n=X), Excluded from analysis (give reasons) (n=X)

SPIRIT
STANDARD PROTOCOL ITEMS: RECOMMENDATIONS FOR INTERVENTIONAL TRIALS

SPIRIT 2013 Checklist: Recommended items to address in a clinical trial protocol and related documents*

Section/item	Item No	Description	Page
**Administrative information**			
Title	1	Descriptive title identifying the study design, population, interventions, and, if applicable, trial acronym	1
Trial registration	2a	Trial identifier and registry name. If not yet registered, name of intended registry	1
	2b	All items from the World Health Organization Trial Registration Data Set	1,2,5,6,7, 8,9,10, 11
Protocol version	3	Date and version identifier	11
Funding	4	Sources and types of financial, material, and other support	1, 12
Roles and responsibilities	5a	Names, affiliations, and roles of protocol contributors	1
	5b	Name and contact information for the trial sponsor	1, 12
	5c	Role of study sponsor and funders, if any, in study design; collection, management, analysis, and interpretation of data; writing of the report; and the decision to submit the report for publication, including whether they will have ultimate authority over any of these activities	12
	5d	Composition, roles, and responsibilities of the coordinating centre, steering committee, endpoint adjudication committee, data management team, and other individuals or groups overseeing the trial, if applicable (see Item 21a for data monitoring committee)	11, 12
**Introduction**			
Background and rationale	6a	Description of research question and justification for undertaking the trial, including summary of relevant studies (published and unpublished) examining benefits and harms for each intervention	2, 3, 4
	6b	Explanation for choice of comparators	7
Objectives	7	Specific objectives or hypotheses	7,8

For peer review only - http://bmjopen.bmj.com/site/about/guidelines.xhtml

Trial design	8	Description of trial design including type of trial (eg, parallel group, crossover, factorial, single group), allocation ratio, and framework (eg, superiority, equivalence, noninferiority, exploratory)	5,6

Methods: Participants, interventions, and outcomes

Study setting	9	Description of study settings (eg, community clinic, academic hospital) and list of countries where data will be collected. Reference to where list of study sites can be obtained	5
Eligibility criteria	10	Inclusion and exclusion criteria for participants. If applicable, eligibility criteria for study centres and individuals who will perform the interventions (eg, surgeons, psychotherapists)	5, 6
Interventions	11a	Interventions for each group with sufficient detail to allow replication, including how and when they will be administered	7
	11b	Criteria for discontinuing or modifying allocated interventions for a given trial participant (eg, drug dose change in response to harms, participant request, or improving/worsening disease)	7
	11c	Strategies to improve adherence to intervention protocols, and any procedures for monitoring adherence (eg, drug tablet return, laboratory tests)	7
	11d	Relevant concomitant care and interventions that are permitted or prohibited during the trial	7
Outcomes	12	Primary, secondary, and other outcomes, including the specific measurement variable (eg, systolic blood pressure), analysis metric (eg, change from baseline, final value, time to event), method of aggregation (eg, median, proportion), and time point for each outcome. Explanation of the clinical relevance of chosen efficacy and harm outcomes is strongly recommended	7, 8, 9, 10
Participant timeline	13	Time schedule of enrolment, interventions (including any run-ins and washouts), assessments, and visits for participants. A schematic diagram is highly recommended (see Figure)	7
Sample size	14	Estimated number of participants needed to achieve study objectives and how it was determined, including clinical and statistical assumptions supporting any sample size calculations	9
Recruitment	15	Strategies for achieving adequate participant enrolment to reach target sample size	5, 9

Methods: Assignment of interventions (for controlled trials)

Allocation:

Sequence generation	16a	Method of generating the allocation sequence (eg, computer-generated random numbers), and list of any factors for stratification. To reduce predictability of a random sequence, details of any planned restriction (eg, blocking) should be provided in a separate document that is unavailable to those who enrol participants or assign interventions	6
Allocation concealment mechanism	16b	Mechanism of implementing the allocation sequence (eg, central telephone; sequentially numbered, opaque, sealed envelopes), describing any steps to conceal the sequence until interventions are assigned	6
Implementation	16c	Who will generate the allocation sequence, who will enrol participants, and who will assign participants to interventions	6
Blinding (masking)	17a	Who will be blinded after assignment to interventions (eg, trial participants, care providers, outcome assessors, data analysts), and how	7
	17b	If blinded, circumstances under which unblinding is permissible, and procedure for revealing a participant's allocated intervention during the trial	7
**Methods: Data collection, management, and analysis**			
Data collection methods	18a	Plans for assessment and collection of outcome, baseline, and other trial data, including any related processes to promote data quality (eg, duplicate measurements, training of assessors) and a description of study instruments (eg, questionnaires, laboratory tests) along with their reliability and validity, if known. Reference to where data collection forms can be found, if not in the protocol	9, 10, 11
	18b	Plans to promote participant retention and complete follow-up, including list of any outcome data to be collected for participants who discontinue or deviate from intervention protocols	9, 10, 11
Data management	19	Plans for data entry, coding, security, and storage, including any related processes to promote data quality (eg, double data entry; range checks for data values). Reference to where details of data management procedures can be found, if not in the protocol	6, 11
Statistical methods	20a	Statistical methods for analysing primary and secondary outcomes. Reference to where other details of the statistical analysis plan can be found, if not in the protocol	10
	20b	Methods for any additional analyses (eg, subgroup and adjusted analyses)	10
	20c	Definition of analysis population relating to protocol non-adherence (eg, as randomised analysis), and any statistical methods to handle missing data (eg, multiple imputation)	10

For peer review only - http://bmjopen.bmj.com/site/about/guidelines.xhtml

Methods: Monitoring

Data monitoring	21a	Composition of data monitoring committee (DMC); summary of its role and reporting structure; statement of whether it is independent from the sponsor and competing interests; and reference to where further details about its charter can be found, if not in the protocol. Alternatively, an explanation of why a DMC is not needed	11
	21b	Description of any interim analyses and stopping guidelines, including who will have access to these interim results and make the final decision to terminate the trial	10
Harms	22	Plans for collecting, assessing, reporting, and managing solicited and spontaneously reported adverse events and other unintended effects of trial interventions or trial conduct	8, 11
Auditing	23	Frequency and procedures for auditing trial conduct, if any, and whether the process will be independent from investigators and the sponsor	11

Ethics and dissemination

Research ethics approval	24	Plans for seeking research ethics committee/institutional review board (REC/IRB) approval	2, 11
Protocol amendments	25	Plans for communicating important protocol modifications (eg, changes to eligibility criteria, outcomes, analyses) to relevant parties (eg, investigators, REC/IRBs, trial participants, trial registries, journals, regulators)	8, 9
Consent or assent	26a	Who will obtain informed consent or assent from potential trial participants or authorised surrogates, and how (see Item 32)	5
	26b	Additional consent provisions for collection and use of participant data and biological specimens in ancillary studies, if applicable	5, 9
Confidentiality	27	How personal information about potential and enrolled participants will be collected, shared, and maintained in order to protect confidentiality before, during, and after the trial	11
Declaration of interests	28	Financial and other competing interests for principal investigators for the overall trial and each study site	12
Access to data	29	Statement of who will have access to the final trial dataset, and disclosure of contractual agreements that limit such access for investigators	12
Ancillary and post-trial care	30	Provisions, if any, for ancillary and post-trial care, and for compensation to those who suffer harm from trial participation	9, 11

For peer review only

Dissemination policy	31a	Plans for investigators and sponsor to communicate trial results to participants, healthcare professionals, the public, and other relevant groups (eg, via publication, reporting in results databases, or other data sharing arrangements), including any publication restrictions	11
	31b	Authorship eligibility guidelines and any intended use of professional writers	12
	31c	Plans, if any, for granting public access to the full protocol, participant-level dataset, and statistical code	11
**Appendices**			
Informed consent materials	32	Model consent form and other related documentation given to participants and authorised surrogates	Consent Form attached
Biological specimens	33	Plans for collection, laboratory evaluation, and storage of biological specimens for genetic or molecular analysis in the current trial and for future use in ancillary studies, if applicable	Consent Form attached

*It is strongly recommended that this checklist be read in conjunction with the SPIRIT 2013 Explanation & Elaboration for important clarification on the items. Amendments to the protocol should be tracked and dated. The SPIRIT checklist is copyrighted by the SPIRIT Group under the Creative Commons "Attribution-NonCommercial-NoDerivs 3.0 Unported" license.

For peer review only
